# Self‐reported alcohol consumption of pregnant women and their partners correlates both before and during pregnancy: A cohort study with 21,472 singleton pregnancies

**DOI:** 10.1111/acer.14806

**Published:** 2022-05-15

**Authors:** Taija Voutilainen, Jaana Rysä, Leea Keski‐Nisula, Olli Kärkkäinen

**Affiliations:** ^1^ 163043 School of Pharmacy Faculty of Health Sciences University of Eastern Finland Kuopio Finland; ^2^ 60650 Department of Obstetrics and Gynecology Kuopio University Hospital Kuopio Finland; ^3^ 163043 Institute of Clinical Medicine University of Eastern Finland Kuopio Finland

**Keywords:** alcohol, alcohol dependence, fetal alcohol spectrum disorders, partners, pregnancy

## Abstract

**Background:**

The partners’ role in determining the alcohol consumption behavior of pregnant women is not well studied. We measured alcohol use before and during pregnancy in pregnant women and their partners to evaluate the correlation in their levels of consumption.

**Methods:**

We evaluated the self‐reported alcohol use of 14,822 women and their partners during 21,472 singleton pregnancies delivered in Kuopio University Hospital, Finland during the period 2009‒2018. The information was gathered during pregnancy and at the time of childbirth and recorded in two databases that were merged to yield a single cohort. Missing data were accounted for by multiple imputation using the predictive mean matching method.

**Results:**

In 86% of the pregnancies, women reported alcohol use before pregnancy, whereas in 4.5% of the pregnancies women reported alcohol use during pregnancy. In contrast, no decrease was detected in their partners’ alcohol use before or during pregnancy. In 26% of the pregnancies, the woman reported stopping alcohol use only after recognizing that she was pregnant. Before pregnancy, there were strong correlations between the pregnant women and their partners in the total Alcohol Use Disorders Identification Test score (*r*_s_ = 0.69, *p* < 0.0001) and the self‐reported average weekly amount of alcohol consumed (*r*_s_ = 0.56, *p* < 0.0001). During pregnancy, there were weak correlations between the pregnant women and their partners in the frequency of drinking (*r*_s_ = 0.20, *p* < 0.0001) and the average weekly amount of alcohol consumed (*r*_s_ = 0.18, *p* < 0.0001).

**Conclusions:**

The self‐reported alcohol consumption of pregnant women and their partners was positively correlated both before and during pregnancy, though the correlation declined substantially during pregnancy. Evaluating the alcohol consumption of both parents before pregnancy could assist in identifying women at risk of prenatal alcohol exposure. Supporting a reduction in partners' alcohol use could help to reduce pregnant women's alcohol consumption and prevent its associated harms.

## Graphical abstract

In a cohort of 21,472 singleton pregnancies, self‐reported alcohol consumption of women and their partners was positively correlated, both before and during pregnancy. This indicates that (a) evaluating the partners' alcohol use before pregnancy might enhance the ability of maternity clinics to identify women who are at risk of using alcohol during their pregnancy, and (b) reducing the alcohol use of both parents before pregnancy might help in reducing the harms related to prenatal alcohol exposure.

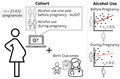

## INTRODUCTION

Alcohol is a known teratogen and alcohol use during pregnancy remains a significant public health problem. The estimated proportion of women consuming alcohol during pregnancy varies greatly between different countries; the global estimate being 10%, while in Europe nearly 16% of women report consuming alcohol at least to some extent during their pregnancy (Mårdby et al., 2017; Popova et al., 2017). Prenatal alcohol exposure is known to impair fetal health without any well‐defined safe level (Day et al., 2013; Dejong et al., 2019; Mårdby et al., 2017). Alcohol use can lead to stillbirths, different kinds of fetal malformations, affect child's psychological and physical growth after birth, or the development of fetal alcohol spectrum disorders (FASD) (Barr et al., 2006; Cornelius et al., 2002; Day et al., 2013; Dejong et al., 2019; Hoyme et al., 2016; Jacobson et al., 2021; Jacobson & Jacobson, 1999; Jones et al., 1973; Mårdby et al., 2017; Streissguth et al., 1990, 1994). FASD are a group of neurodevelopmental conditions and the most important nongenetic cause of cognitive disability worldwide (Dejong et al., 2019). Because of these lifelong and prevalent consequences on individual health, alcohol use during pregnancy represents a burden to the individuals as well as to society at large.

When studying alcohol use in relation to pregnancy, it is women who have predominantly been the object of research. Consequently, it is known that alcohol use before pregnancy predicts maternal drinking behavior during pregnancy (Corrales‐Gutierrez et al., 2020; Skagerstróm et al., 2011). In addition, women using alcohol more frequently (Young‐Wolff et al., 2020), or reporting binge drinking before pregnancy (Ethen et al., 2009), are more likely to also continue alcohol use during their pregnancy.

Meanwhile, there are very few studies that have investigated the effect of the pregnant women's partners’ alcohol consumption or the partners’ influence on the alcohol consumption of the pregnant women. The available studies indicate that the drinking behavior of women and their partners are positively correlated (Corrales‐Gutierrez et al., 2020; Rubin et al., 1988) and that the level of spousal drinking is associated with maternal drinking during pregnancy (Bakhireva et al., 2011; Kautz‐Turnbull et al., 2021). In each of these studies, several hundred pregnant women were interviewed. Moreover, there is a lack of studies that would have investigated if there are associations between alcohol use of pregnant women and their partners both before and during pregnancy. In animal studies, male exposure to alcohol during the time of periconception seems to affect the offspring (Abel, 2004). Consequently, it is important to study the before‐pregnancy alcohol use because this is linked to unintentional fetal exposure during early pregnancy.

Therefore, our aims were to (1) investigate the alcohol use of the pregnant women and their partners both before and during pregnancy; (2) explore the associations between the use before and during pregnancy; and (3) examine if there were correlations between the alcohol use of the pregnant women and their partners. To answer these questions, we compiled a large Finnish birth cohort that included information on 14,822 individual women and their partners in 21,472 singleton pregnancies.

## MATERIALS AND METHODS

The cohort consisted of 21,472 singleton pregnancies lasting at least 22 full gestational weeks, with deliveries between January 1st, 2009, and May 31st, 2018, in Kuopio University Hospital, Finland. Information about both pregnant women and their partners was gathered during pregnancy via electronic questionnaires, but also in regular maternity clinic visits and at the time of birth, in the maternity hospital. In the first maternity‐clinic visit (usually before the 10th gestation week), the women were provided with login information into the electronic questionnaire that inquired about the alcohol use of themselves and their spouses. The women answered these online questions during their pregnancy, and the answers were restored into a database called PikkuHaikara. The information reported by the health care professionals in the maternity hospital were recorded into a second database called Haikara.

The information gathered from both parents included their age during the pregnancy, and their alcohol use both before and during pregnancy. In the online questionnaire, a ten‐question Alcohol Use Disorders Identification Test (AUDIT) was used to evaluate the alcohol‐use risk level 1 year prior to the pregnancy. In the Finnish AUDIT questionnaire, binge drinking was defined as having six or more alcohol doses on one occasion. One alcohol dose was defined to contain 12 g of pure EtOH. Furthermore, the average weekly alcohol doses both before and during pregnancy and the frequency of drinking during pregnancy were inquired.

In addition, maternal parity, gravidity, marital status (both during pregnancy and at the time of the childbirth), body weight, height, body mass index in the first trimester, and weight gain during the pregnancy were recorded. The birth outcomes were documented by the health care professionals at the maternity hospital including the duration of the pregnancy at delivery, birth weight, head circumference, fetal sex, and Apgar scores (1 and 5 min) of the birth, as well as recognized birth defects, and stillbirths with the length of the umbilical cord and placental weight also being measured. Furthermore, the duration of the hospital stay after delivery and any possible neonatal treatments were documented.

### Statistical analysis

**FIGURE 1:**
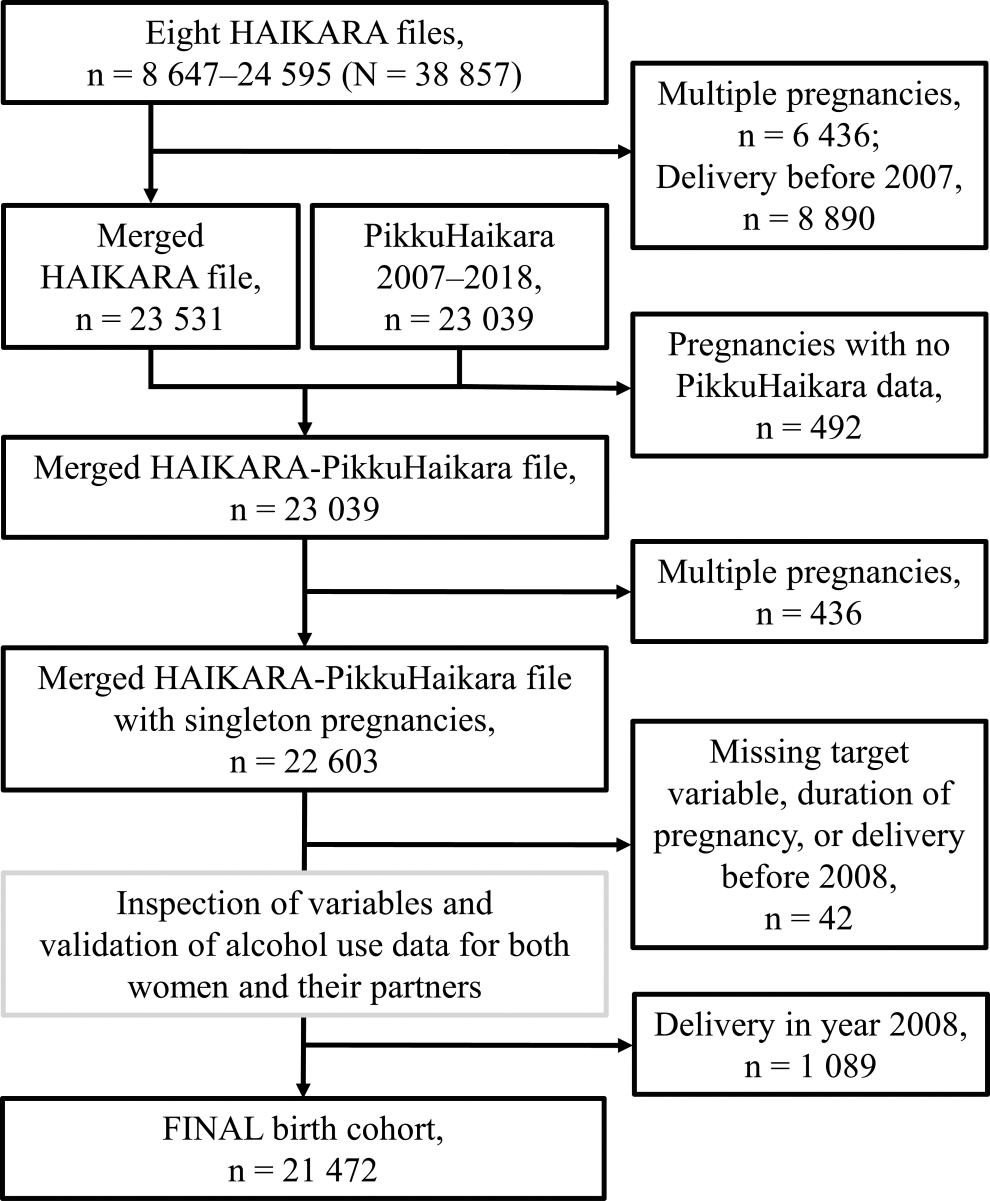
Compilation of the birth cohort and exclusion criteria. The cohort was compiled from two databases: (1) HAIKARA, data reported by the health care professionals in the maternity hospital and (2) PikkuHaikara, data self‐reported by the pregnant women using an electronic questionnaire

IBM SPSS Statistics Software (v. 27.0.1.0) was used in both data management and statistical analysis. The two data files (PikkuHaikara and Haikara) were merged into a common database by matching the cases by the pregnancy identification code (Figure 1). The alcohol consumption variables were cross‐validated to minimize the possibility of conflicting information from the individual parents. In addition, outliers were inspected, and impossibly high or low values were deleted.

The missing data were accounted for by undertaking multiple imputation (MI), which not only fully utilizes the collected data and thus increases the statistical power but also takes into consideration the uncertainty of the imputed values (Graham, 2009; Grigsby & Mclawhorn, 2019; Van Ginkel et al., 2019). In this study, we used MI by Chained Equation with a predictive mean matching method (*M* = 40, *k* = 5). This method applies regression analysis to find a pool of cases (*k*‐nearest values, here *k* = 5) that closely resemble a case with a missing value (Kleinke, 2017). From that pool of cases, it then randomly selects one case whose value it imputes in the place of the missing value. For more information on MI, the equations used, the sensitivity analysis conducted, and the MI models please see the Methods S1.

However, while MI is well suited for estimates (mean and standard deviation) and correlations, it cannot be used to describe the frequencies or proportions (Graham, 2009). Thus, the reported frequencies and percentages are those present in the original dataset. The mean and standard deviation were analyzed both for available cases and for the multiple imputed datasets. To yield the final estimates from the multiple imputed data, Rubin's Rules were used to combine the parameter estimates in each of the complete datasets (*M* = 40) (Rubin, 1987).

Bivariate nonparametric correlations of alcohol use variables were tested using Spearman's rho. To account for the inflation of a false positive rate in multiple testing, the preset two‐tailed 0.05 alpha level was divided by the number of correlations (*n* = 91). Thus, correlation coefficients with a *p*‐value less than the corrected alpha (0.0005) were considered statistically significant (Bonferroni's method). Finally, we conducted a sensitivity analysis to evaluate whether the nonindependence of the pregnancy cases in the original cohort (*n* = 21,472) affected our results. We randomly selected one pregnancy for each woman, and then compared these independent‐case results (*n* = 14,822) to the results of all pregnancies in the cohort.

## RESULTS

Our cohort is representative of the pregnant and parenting population in Finland. During the study period (2009‒2018), 63% of the 14,822 women gave birth once, while 37% gave birth two or more times (Table 1). In 42% of the 21,472 pregnancies, the woman was nulliparous, and about 5% of all pregnancies were conceived with assisted reproductive technologies. In 82% of the pregnancies, the women were in a relationship (i.e., engaged, married, or cohabiting with a partner) at the time of the childbirth. On average, the partners were slightly older than the women. The mean duration of the gestation at the time of the delivery was 277 ± 14 days (mean ± SD, Table 2); 6% of the babies were born preterm (duration of gestation less than 259 days). Nearly all babies were born alive and in hospital. The sex ratio of the babies was even: 51% were males and 49% were females. About 15% of the newborns required some form of special treatment immediately after birth in the labor room, in the ward, or in the neonatal intensive care unit.

**TABLE 1:** Demographic data of the parents

Characteristics	Valid *n* ^a^	% of all cases	Mean ± SD	Range
Women	14,822	100.0^b^		
Age (years during pregnancy)	21,472	100.0	29.8 ± 5.4	15 to 54
≤25	4735	22.1		
26 to 29	5945	27.7		
30 to 33	5632	26.2		
≥34	5160	24.0		
Gravidity	21,472	100.0	2.6 ± 1.9	1 to 20
Parity	21,472	100.0	1.1 ± 1.4	0 to 16
Nulliparous	9037	42.1		
Primiparous	6835	31.8		
Multiparous	5600	26.1		
Number of births^c^			1.4 ± 2.2	1 to 7
1	9337	63.0^b^		
2 or more	5485	37.0^b^		
Used ART	1009	4.7		
Marital status (at childbirth)	21,338	99.4		
In a relationship^d^	17,564	81.8		
Single^e^	3774	17.6		
BMI before pregnancy (kg/m^2^)	20,493	95.4	24.8 ± 5.1	13.5 to 57.2
Partners				
Age (years during pregnancy)	17,535	81.7	31.5 ± 6.4	15 to 67
Unknown	3937	18.3		
≤25	2956	13.8		
26 to 29	4149	19.3		
30 to 33	4635	21.6		
≥34	5795	27.0		

Note

*N* = 21,472. The mean and standard deviation estimate for variables with missing values (% of all cases less than 100) are based on multiple imputation.

Abbreviations: ART, assisted reproductive technologies; BMI, body mass index.

^a^ The number of cases with information in the original data.

^b^ The percentage refers to the percentage of women in the cohort.

^c^ Number of births per mother during the cohort time period.

^d^ Married, engaged, or living with a partner.

^e^ Single, widowed, or not living with a partner.

**TABLE 2:** Details of the pregnancies and the birth outcomes

Characteristics	Valid *n* ^a^	% of all cases	Mean ± SD
Duration of pregnancy (days)	21,472	100	277.2 ± 14.3
Birthweight (g)	21,464	~100	3477.6 ± 577.9
Head circumference (cm)	20,668	95.8	34.9 ± 2.2
Umbilical cord length (cm)	21,239	98.9	59.7 ± 13.8
Placental weight (g)	21,179	98.6	598.0 ± 131.7
Hospital stay after delivery (days)	21,472	100	3.7 ± 2.8
Apgar^b^			
1 min	21,470	100	8.6 ± 1.2
5 min	21,470	100	8.9 ± 1.1

Characteristics	Specification	Valid *n* ^a^	% of all cases	Specification	Valid *n* ^a^	% of all cases
Fetal sex^c^	Male	10,861	50.6	Female	10,609	49.4
Born alive	Yes	21,403	99.7	No	69	0.3
Birth defects	Yes	86	0.4	No	21,386	99.6
Required neonatal treatment^d^	Yes	3304	15.4	No	18,168	84.6
Born in hospital	Yes	21,376	99.6	No	91	0.4

Note

*N* = 21,472. The mean and standard deviation estimates for variables with missing values (% of all cases less than 100) are based on multiple imputation.

^a^ The number of cases with the information in the original data.

^b^ The mean Apgar value was used in multiple imputation.

^c^ The sex was unknown for two prematurely born babies.

^d^ Some form of specialized treatments, that is, resuscitation and/or assistance in breathing, treatment for hypoglycemia, antibiotics, phototherapy, and/or other types of intensive care were given to the baby either during its birth, in the ward or in the neonatal intensive care unit.

**FIGURE 2:**
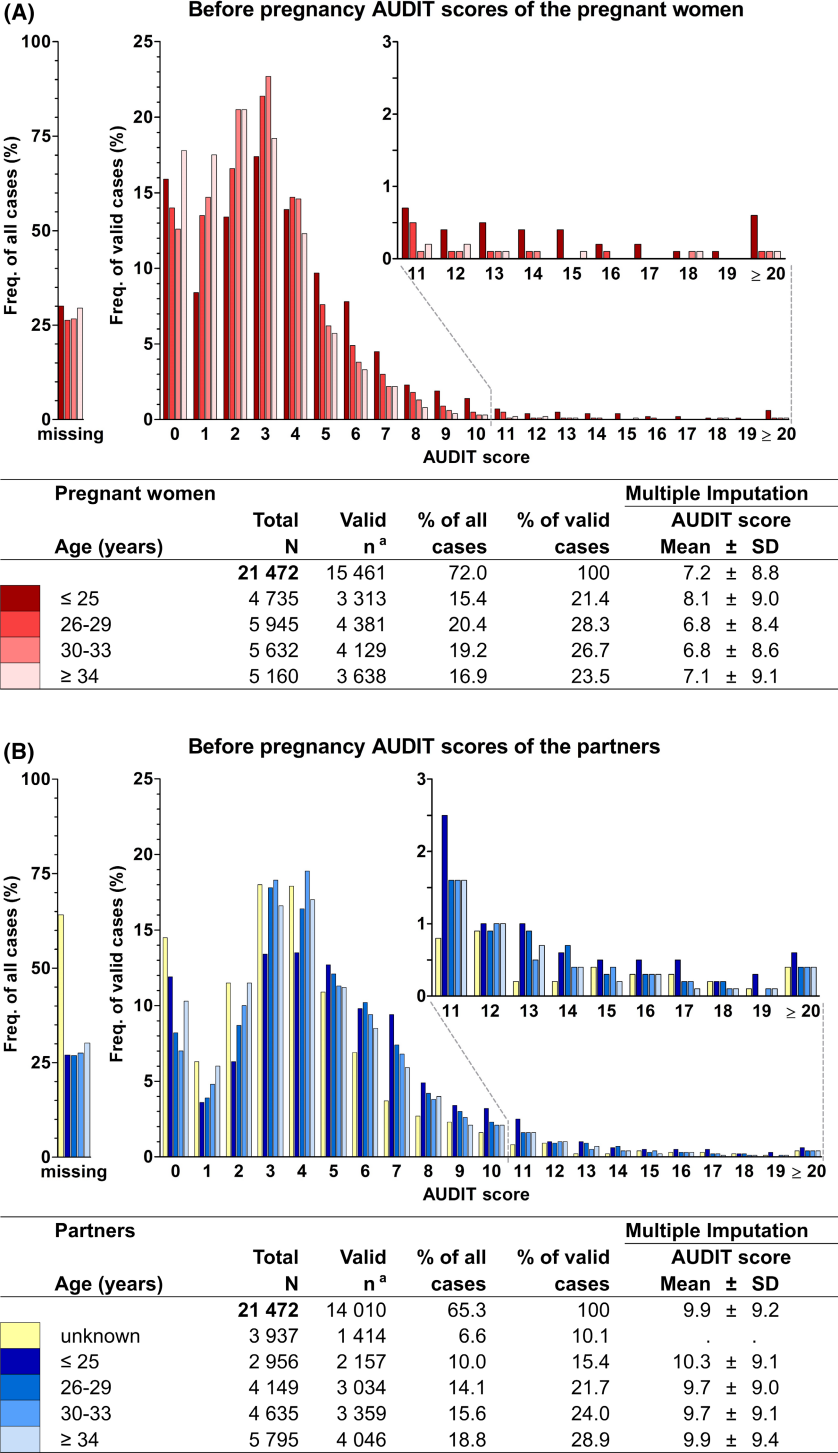
Total AUDIT score distribution and mean within age groups in women (A) and their partners (B). *N* = 21,472 pregnancies. The AUDIT test evaluated alcohol use 1 year prior to the pregnancy. The total AUDIT score, that is, the total of the ten AUDIT questions was missing if one or more of the questions was missing. Note that multiple imputation (MI) cannot be used to report frequencies. Thus, the frequency of all cases bar graphs show what portion of the total score was missing in each of the age groups in the original data. The bars in the valid cases graphs represent 100% of the number of valid cases in the original data ^a^ in each age group. The estimates of AUDIT scores multiple imputed mean and standard deviation are presented along with the age group color coding for the bar graphs. In women, the youngest age group engaged in the riskiest drinking behavior represented by more frequent high AUDIT scores in the original data and the highest mean values in the multiple imputed data. In the partners, the differences in total AUDIT scores among age groups were more subtle. It should be noted that there is a large proportion of partners whose age is unknown in the original data, but since also the age variable was multiple imputed, there is no mean estimate for this group in the multiple imputed data

**FIGURE 3:**
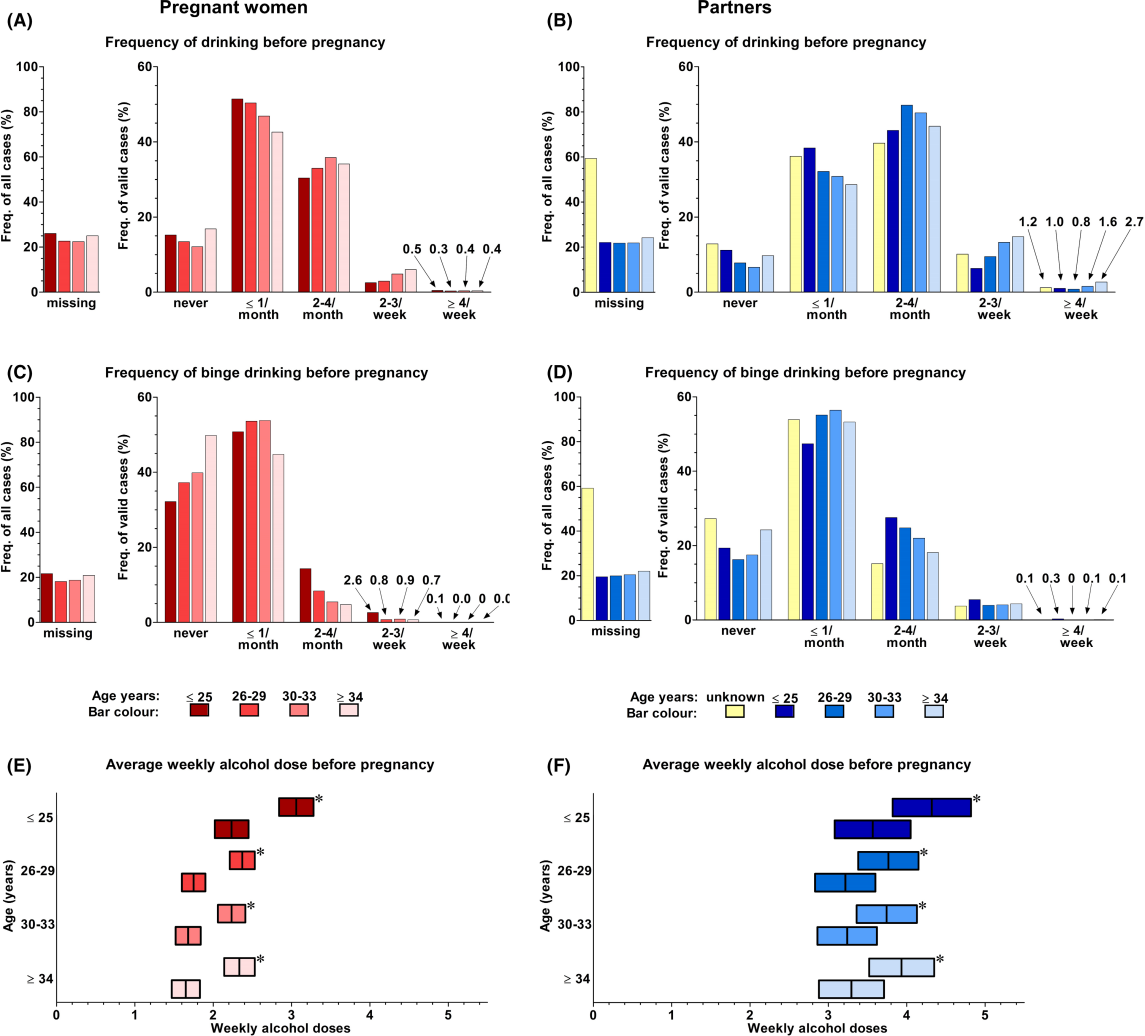
Before‐pregnancy alcohol use of the parents in the different age groups. *N* = 21,472 pregnancies. The bar graphs represent the frequency of drinking before pregnancy in A women (*n* of valid cases 16,340) and B partners (*n* of valid cases 15,162) and the frequency of binge drinking before pregnancy in C women (*n* of valid cases 17,233) and D partners (*n* of valid cases 15,515) in the original data. Note that multiple imputation (MI) cannot be used to report frequencies. Thus, the frequency of all cases bar graphs shows the proportion of the pregnancy cases in each of the age groups that had a missing answer in the original data while the frequency of valid cases bar graphs illustrates the distribution of the valid answers in each age group (each age group gives a total of 100%) in the original data. In panels E and F, the bars represent the multiple imputed mean and 99% confidence intervals of the before‐pregnancy average weekly alcohol dose of women and their partners, respectively. The bars with an asterisk (*) in the top right corner of the bar are the estimates of the alcohol users, that is, those having one or more weekly doses. Bars without an asterisk are those of all subjects in each age group. In both parents, the youngest age group used alcohol before pregnancy less frequently, but binge drank more frequently than the older age groups. The women, in general, used alcohol less than their partners

The self‐reported alcohol consumption were compared to contrast the women with their partners and to evaluate the change in alcohol use from before to during pregnancy. The AUDIT questionnaire evaluated alcohol use 1 year prior to the pregnancy. Due to missing values in the original data, the total AUDIT score was missing in about every third case in both women and their partners (Figure 2). Of those with a valid final score in the original data, about 7% of women and 14% of partners had a score indicating an increased alcohol‐use risk level, that is, a score above 7 in women or a score above 8 in partners, respectively (Babor et al., 2001) (data not shown). The multiple imputed means and standard deviations of the AUDIT scores were 7 ± 9 for the women and 10 ± 9 for the partners (mean ± SD, Figure 2). The frequency of drinking before pregnancy and frequency of binge drinking were questions inquired in the AUDIT questionnaire (Figure 3). The average weekly alcohol doses before pregnancy were about 1.5 doses less in women than the amounts consumed by their partners.

**FIGURE 4:**
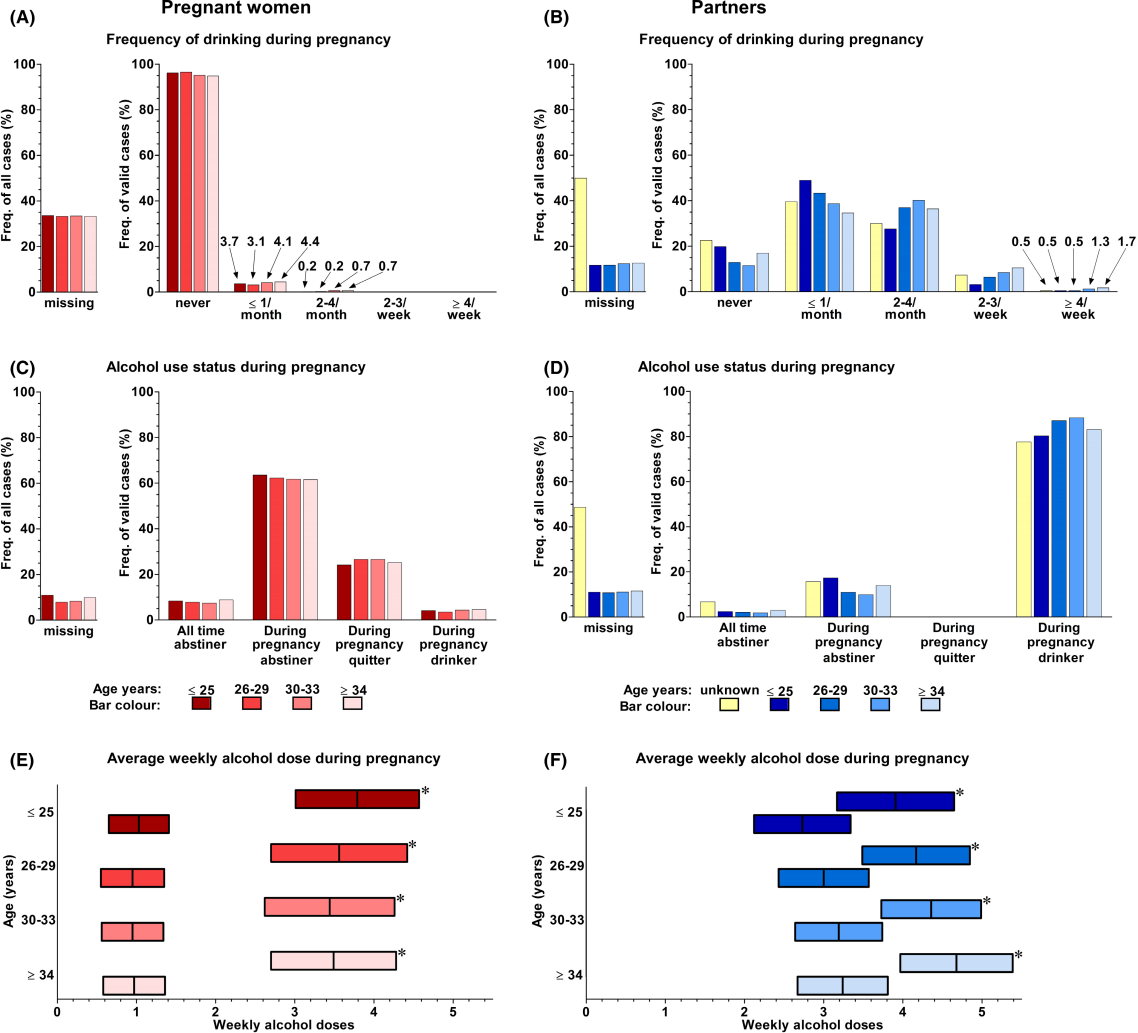
During‐pregnancy alcohol use of the parents in the different age groups. *N* = 21,472 pregnancies. The bar graphs represent the frequency of drinking during pregnancy in A women (*n* of valid cases 14,301) and B partners (*n* of valid cases 17,371) and the during‐pregnancy alcohol use status of C women (*n* of valid cases 19,509) and D partners (*n* of valid cases 17,601). Note that multiple imputation (MI) cannot be used to report frequencies. Thus, the frequency of all cases bar graphs shows what proportion of the pregnancy cases in each of the age groups had a missing answer in the original data while the frequency of valid cases bar graphs illustrates the distribution of the valid answers in each age group (each age group gives a total of 100%). In panels E and F, the bars represent the multiple imputed mean and its 99% confidence interval of the during‐pregnancy average weekly alcohol dose of women and their partners, respectively. The bars with the asterisk (*) in the top right corner of the bar are the estimates of the alcohol users, that is, those having one or more weekly doses. Bars without an asterisk represent all subjects in each age group. In summary, in about 95% of the pregnancies, the women reported never having consumed alcohol during pregnancy. Although, in about one‐quarter of pregnancies, the women reported stopping alcohol use only after becoming aware that they were pregnant which indicates possible prenatal alcohol exposure during early pregnancy. None of the partners reported quitting alcohol use during their partner's pregnancy. The women, in general, used substantially less alcohol during their pregnancy than their partners

In general, the women reduced or terminated their alcohol use during pregnancy, while there was no clear change in alcohol use by their partners (Figures 3 and 4). In 4.5% of pregnancies, women still reported using alcohol during pregnancy. In contrast, in 86% of pregnancies, women had been using alcohol before pregnancy. However, in about 26% of the pregnancies, the women reported they had stopped using alcohol after pregnancy recognition (Figure 4C). In 84% of the pregnancies, the women's partners used alcohol during pregnancy, a value slightly reduced from the 91% who had consumed alcohol before their partner became pregnant (Figures 3 and 4). However, none of the partners reported stopping alcohol use during the pregnancy (Figure 4D). Furthermore, the mean weekly self‐reported alcohol dose of women who reported consuming one or more doses during pregnancy (3.6; 99% CI 2.8 to 4.3), was substantially higher than the mean weekly self‐reported alcohol dose of women who reported consuming one or more doses before pregnancy (2.5; 99% CI 2.3 to 2.6).

When comparing the different age groups in women, the youngest age group (aged 25 years or less) had the riskiest alcohol drinking patterns before pregnancy. In the original data, they had more frequently high total AUDIT scores, that is, a score above 7 (Figure 2), but they also reported more frequent bouts of binge drinking (Figure 3). In addition, in the multiple imputed data, the mean total AUDIT score (8.1 ± 9.0) (Figure 2) and mean self‐reported weekly alcohol dose before pregnancy (3.1; 99% CI 2.8 to 3.3 for those who used alcohol) (Figure 3) in the youngest age group were the highest. However, when they became pregnant, the drinking behavior of younger women resembled that of the older age groups (Figure 4).

**FIGURE 5:**
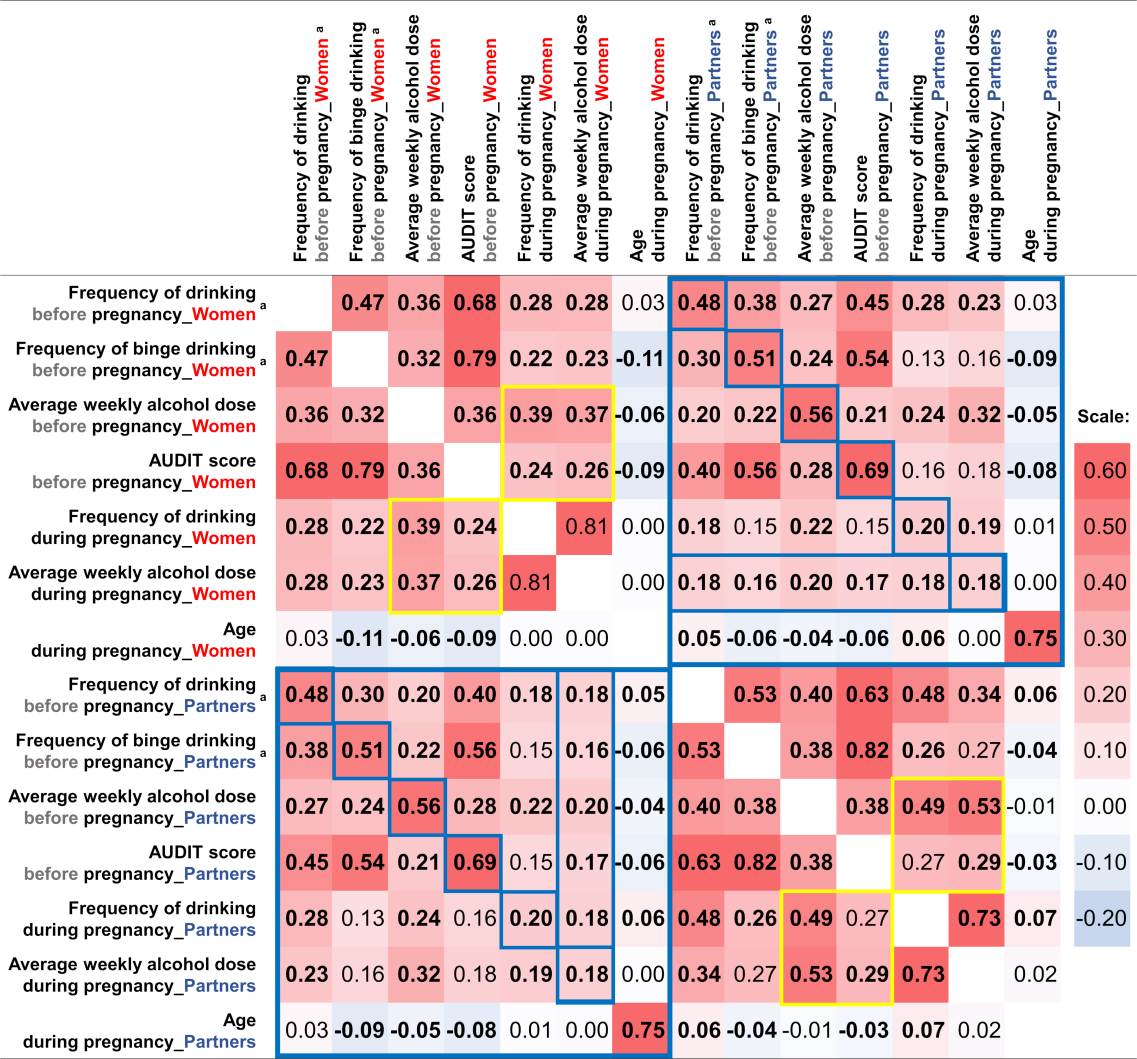
Correlation heatmap of the self‐reported alcohol use and the age variables of the women and their partners. The Spearman rho's correlation coefficients are those of the multiple imputed data (MI = 40). Thus, the *N* in each cell is 21,472. Statistically significant correlations (*p*‐value below multiple testing corrected alpha = 0.0005) are indicated in bold. The largest dark blue rectangles highlight the correlations between the variables of the women and their partners, and the smaller rectangles inside the blue rectangles show the correlations between the same set of alcohol use questions. The yellow rectangles in the upper left corner and the lower right corner highlight the correlations between the before‐ and during‐pregnancy in women and their partners, respectively. ^a^The before‐pregnancy frequency of drinking and frequency of binge drinking are both questions in the AUDIT questionnaire, which explains the stronger correlation between them and the total AUDIT score than the rest of the variables. The alcohol use of the parents is similar to each other: There was a strong statistically significant positive correlation between the parents’ before‐pregnancy alcohol use variables and a weak statistically significant positive correlation between the parents’ during‐pregnancy alcohol use variables

Alcohol use before pregnancy correlated significantly and positively with its use during pregnancy in both women and their partners (Figure 5). In the mothers, the before‐pregnancy average weekly alcohol dose and the total AUDIT score were moderately associated with both the frequency of drinking during pregnancy (*r*_s_ = 0.39, *p* < 0.0001; *r*_s_ = 0.24, *p* = 0.0003, respectively) and the average weekly alcohol dose during pregnancy (*r*_s_ = 0.37, *p* < 0.0001; *r*_s_ = 0.26, *p* < 0.0001, respectively). These correlations were stronger in their partners, although the correlation between the total AUDIT score and frequency of drinking during pregnancy had a p‐value above the multiple testing corrected alpha level of 0.0005 (*r*_s_ = 0.27, *p* = 0.003).

Moreover, the alcohol use of pregnant women and their partners was similar (Figure 5). Strong positive correlations were observed between the pregnant women and their partners in their frequency of drinking before pregnancy (*r*_s_ = 0.48, *p* < 0.0001), and also in the frequency of binge drinking before pregnancy (*r*_s_ = 0.51, *p* < 0.0001), average weekly alcohol dose before pregnancy (*r*_s_ = 0.56, *p* < 0.0001), as well as in the total AUDIT score (*r*_s_ = 0.69, *p* < 0.0001). The correlations between the during‐pregnancy alcohol use of the parents were weaker than those observed before pregnancy. The correlations of the frequency of drinking during pregnancy and the average weekly alcohol dose during pregnancy between the parents were *r*_s_ = 0.20 (*p* < 0.0001) and *r*_s_ = 0.18 (*p* < 0.0001), respectively. Notably, all the alcohol‐use questions of the partners displayed a weak statistically significant association with the during‐pregnancy average weekly alcohol dose of the pregnant women.

Finally, we compared the multiple imputed results to those of the available case analysis (pairwise deletion) from the original data and the results of the sensitivity analysis. In the original data, the mean of the total AUDIT scores for both parents was twice lower than the multiple imputed estimate (Table S1). There were no substantial differences between the average weekly alcohol dose estimates in the original and the multiple imputed datasets. However, the mean average weekly alcohol dose of women was about one unit dose lower in the original data than the estimate in the multiple imputed estimates. The direction of the correlations in the original data was the same as in the multiple imputed data, but there were major differences in their magnitude (Figure S1). The sensitivity analysis showed that the results were similar between the item and total score level multiple imputations (Table S2 and Figure S2). Moreover, the results from our analysis, where all the pregnancies from the cohort were included, were similar to those obtained by analyzing an independent‐case sample, where one pregnancy was selected randomly for each woman in the cohort (*n* = 14,822) (Table S3, and Figures S3 and S4).

## DISCUSSION

In our study, we found that most women self‐reported quitting alcohol use either before or during early pregnancy, while their partners generally did not change their alcohol use during their partner's pregnancy. Moreover, we found that the self‐reported alcohol use of women and their partners before and during pregnancy was significantly correlated.

In our cohort, less than 5% of pregnant women self‐reported alcohol use during pregnancy, which is in line with other studies, where the prevalence of women using alcohol during pregnancy has been in a range of 6%‒25% in the Western World (Alvik et al., 2006; Ní Shúilleabháin et al., 2013; Nilsen et al., 2008; Pryor et al., 2017; Waterson et al., 1990; Young‐Wolff et al., 2020). However, about one‐fourth of the women reported quitting alcohol use only after they became aware that they were pregnant according to their responses to the question about alcohol‐use status during pregnancy, indicating possible prenatal alcohol exposure during early pregnancy. This agrees with previous reports where the majority of women have ceased drinking only after the recognition of pregnancy, even in those cases where the pregnancy was planned (Alvik et al., 2006; Pryor et al., 2017). Our results indicate that one needs to have a diverse set of questions to reliably estimate the prevalence of prenatally alcohol‐exposed babies. It has been observed that women have reported higher during‐pregnancy alcohol use levels when asked retrospectively (Jacobson et al., 1991). Therefore, our results might underestimate the extent of prenatal alcohol exposure, because the questionnaire was delivered and responded by the women during their pregnancy.

Moreover, binge drinking prior to pregnancy recognition is prevalent (Alvik et al., 2006; Denny et al., 2019; Dumas et al., 2017; Ethen et al., 2009; McCormack et al., 2017) especially among younger women (Pryor et al., 2017). Together with late pregnancy recognition, this has been associated with a risk of FASD in children (May et al., 2014). In our cohort, about one‐tenth of women reported regular, at least twice a month, binge drinking during the year prior to pregnancy. Furthermore, this was more frequent among the women aged 25 years or less. In addition, there was a strong correlation between the binge drinking patterns of pregnant women and their partners. Together these results highlight how important it is that those couples planning pregnancy should be informed on the health hazards posed by alcohol consumption in the early phases of pregnancy. In particular, binge drinking should be avoided because consuming several alcohol doses on a single occasion is more harmful than having the same amount of alcohol consumed over several occasions (Jacobson et al., 1998; Jacobson & Jacobson, 1999). In addition, the accessibility and use of effective contraceptives have been shown to be an effective measure in reducing prenatal alcohol exposure in unintentional pregnancies (Reid et al., 2021).

In our cohort, the rate of decline in drinking during pregnancy in women is comparable to previous reports from Norway and United States (Kitsantas et al., 2014; Mellingen et al., 2013; Roberts et al., 2014). We observed that women who continued drinking alcohol during pregnancy had been generally more heavy drinkers before pregnancy, compared to those who stopped drinking while pregnant. Moreover, in the alcohol drinkers, the before‐ and during‐pregnancy drinking correlated significantly, indicating signs of alcohol dependency and an inability to stop even when pregnant. These results are in line with others reporting that the prepregnancy alcohol use level and binge drinking of the women were important predictors of alcohol use during pregnancy (Chang et al., 2006; Ethen et al., 2009; Skagerstróm et al., 2011; Young‐Wolff et al., 2020). Some earlier studies have claimed that older women are more likely to continue drinking during pregnancy (Mårdby et al., 2017; Nilsen et al., 2008; Pryor et al., 2017). However, we did not observe evidence of this phenomenon in our cohort.

The partners’ alcohol use remained at a similar level both before and during the pregnancy when measured either as the proportion of drinkers or the mean weekly alcohol doses consumed in agreement with an American study (Bailey et al., 2008). In contrast, in Scandinavian countries, there has been a reduction in partners’ alcohol use during pregnancy (Högberg et al., 2016; Mellingen et al., 2013). Since their partner's pregnancy alone was not sufficient to change their drinking habits, it seems that interventions would be needed to help them to cut back or even stop drinking. Previous studies have shown that the partners would be willing to listen to counseling from a midwife on alcohol's detrimental effects (Högberg et al., 2016) and to undertake actions to improve their own health (Shawe et al., 2019).

Furthermore, our findings of a significant positive correlation between before and during pregnancy alcohol drinking between the pregnant women and their partners are in line with the previous literature (May et al., 2014; Rubin et al., 1988; Waterson et al., 1990). Moreover, women seem to be more likely to continue alcohol use during pregnancy if they are living with a heavy drinking partner (Bakhireva et al., 2011) although there are also conflicting reports (Chang et al., 2006). Furthermore, heavy drinking by the pregnant woman's partner has been associated with FASD (May et al., 2014). Overall, the reduction in alcohol use of both parents should be promoted before pregnancy to avoid unintentional prenatal alcohol exposure and to minimize the possibility of the development of FASD (Corrales‐Gutierrez et al., 2020). Counseling both parents represents an opportunity to address their shared norms and attitudes, to make both individuals aware of how damaging exposure to alcohol can be for their fetus, and to highlight the importance of the partner as a support‐giver to the pregnant women (Crawford‐Williams et al., 2015; van der Wulp et al., 2015).

In both women and their partners, the mean multiple imputed AUDIT scores were twice as high as the mean AUDIT scores in the original data. This suggests that those individuals who were heavy drinkers tended not to disclose their alcohol use, which is in line with previous reports of an increased nonresponse rate among heavy alcohol users (Boniface et al., 2017; Jousilahti et al., 2005).

This study has several limitations. First, self‐reported data face limitations related to recall, underreporting, nonresponse, and social desirability biases leading to possible underreporting (Del Boca & Darkes, 2003; Morrow‐Tlucak et al., 1989). Second, the partners’ data were filled in by the women and there was no identification code for the partners. Therefore, we cannot be sure if the same partner is involved in several pregnancies. Third, more options in reporting alcohol drinking habits during pregnancy, such as doses per drinking day and per binge drinking bout, would have improved the data; nonetheless, our approach of offering a multitude of appropriate answer options was likely to both decrease the nonresponse rate and increase the accuracy of reporting (Muggli et al., 2015). Fourth, the women were granted the log‐in information into the online electronic questionnaire at their first maternity‐clinic visit during the first trimester. However, there is no certainty when during their pregnancy the women reported their use. Therefore, from the present data, we cannot make conclusions on whether the duration of the pregnancy affected the reported alcohol use or if there was a difference in use between the trimesters. Lastly, if we had been able to assay biological markers for alcohol consumption, for example, phosphatidyl EtOH measured from whole blood (Finanger et al., 2021), this would have improved the validity of the data.

However, this study has some definitive strengths. First, the cohort was systematically collected and it is a representative sample of the study population, and it also represents well Finnish birth registers (Heino & Gissler, 2020). Our cohort included 21,472 singleton pregnancies that covered more than 95% of the registered 22,557 childbirths (of which some were multiparous) during the cohort time period. Secondly, an electronic questionnaire was used to screen the alcohol use of both women and their partners, reducing the social desirability bias. Thirdly, alcohol use was investigated by a range of questions covering both the time before and that during the pregnancy. Fourth, the sensitivity analysis of the randomly selected one pregnancy independent cases confirmed that although the cohort did not meet the assumption of independence, this did not have a large effect on the results. Instead, by including all 21,472 pregnancies in our analyses we got a more precise picture of the alcohol use during pregnancy in general and a more reliable estimate of prenatally alcohol‐exposed fetuses. Lastly, we utilized MI which takes into account all of the collected data but also the uncertainty of the imputed values, thus generating less bias in the estimates.

## CONCLUSION

In conclusion, in a large and systematically collected representative cohort, we observed that there were significant correlations between the self‐reported alcohol use of pregnant women and their partners both before and during pregnancy. This indicates that evaluating the alcohol use of the pregnant women's partners might improve the identification of those women who are at risk of using alcohol during their pregnancy. Moreover, this could indicate that reducing the partners’ alcohol use before pregnancy might help to reduce the during‐pregnancy alcohol use of their pregnant spouses. Further studies are needed to investigate whether supporting the reduction of alcohol use of both women and their partners before pregnancy could reduce drinking during early pregnancy and unintentional prenatal alcohol exposure.

## CONFLICT OF INTEREST

None.

## AUTHOR CONTRIBUTIONS

TV compiled the databases into a single cohort, performed data management and data analysis, and wrote the first draft of the manuscript. LK‐N provided the epidemiological database. JR and OK supervised the study. All authors have read and approved the final manuscript.

## ETHICAL APPROVAL

This study is a part of the Kuopio Birth Cohort study (KuBiCo, www.kubico.fi) (Huuskonen et al., 2018), which has received approval from the Research Ethics Committee of Hospital District of Central Finland.

## Supporting information

Fig S1

Fig S2

Fig S3

Fig S4

Table S1

Table S2

Table S3

Method S1

Supplementary Material

## References

[acer14806-bib-0001] Abel, E. (2004) Paternal contribution to fetal alcohol syndrom. Addiction Biology, 9, 127–133. 15223537 10.1080/13556210410001716980

[acer14806-bib-0002] Alvik, A. , Heyerdahl, S. , Haldorsen, T. & Lindemann, R. (2006) Alcohol use before and during pregnancy: a population‐based study. Acta Obstetricia et Gynecologica Scandinavica, 85, 1292–1298. 17091405 10.1080/00016340600589958

[acer14806-bib-0055] Babor, T. , Higgins‐Biddle, J. , Saunders, J. & Montiero, M. (2001) AUDIT: the Alcohol Use Disorders Identification Test: Guidelines for Use in Primary Care (second edition). 2nd, Geneve, Switzerland: World Health Organization.

[acer14806-bib-0003] Bailey, J. , Hill, K. , Hawkins, J. , Catalano, R. & Abbott, R. (2008) Men’s and women’s patterns of substance use around pregnancy. Birth, 35, 50–59. 18307488 10.1111/j.1523-536X.2007.00211.x

[acer14806-bib-0004] Bakhireva, L.N. , Wilsnack, S.C. , Kristjanson, A. , Yevtushok, L. , Onishenko, S. , Wertelecki, W. et al. (2011) Paternal drinking, intimate relationship quality, and alcohol consumption in pregnant Ukrainian women. Journal of Studies on Alcohol and Drugs, 72, 536–544. 21683035 10.15288/jsad.2011.72.536 PMC3125877

[acer14806-bib-0005] Barr, H. , Bookstein, F. , O'Malley, K. , Connor, P. , Huggins, J. & Streissguth, A. (2006) Binge drinking during pregnancy as a predictor of psychiatric disorders on the structured clinical interview for DSM‐IV in young adult offspring a 25‐year follow‐up. American Journal of Psychiatry, 163, 1061–1065. 16741207 10.1176/ajp.2006.163.6.1061

[acer14806-bib-0006] Boniface, S. , Scholes, S. , Shelton, N. & Connor, J. (2017) Assessment of non‐response bias in estimates of alcohol consumption: applying the continuum of resistance model in a general population survey in England. PLoS One, 12, e0170892. 28141834 10.1371/journal.pone.0170892 PMC5283659

[acer14806-bib-0007] Chang, G. , McNamara, T. , Orav, E. & Wilkins‐Haug, L. (2006) Alcohol use by pregnant women: partners, knowledge, and other predictors. Journal of Studies on Alcohol, 67, 245–251. 16562406 10.15288/jsa.2006.67.245 PMC1540454

[acer14806-bib-0008] Cornelius, M. , Goldschmidt, L. , Day, N. & Larkby, C. (2002) Alcohol, tobacco and marijuana use among pregnant teenagers: 6‐year follow‐up of offspring growth effects. Neurotoxicology and Teratology, 24, 703–710. 12460652 10.1016/s0892-0362(02)00271-4

[acer14806-bib-0009] Corrales‐Gutierrez, I. , Mendoza, R. , Gomez‐Baya, D. & Leon‐Larios, F. (2020) Understanding the relationship between predictors of alcohol consumption in pregnancy: towards effective prevention of FASD. International Journal of Environmental Research and Public Health, 17, 1388. 10.3390/ijerph17041388 PMC7068254 32098098

[acer14806-bib-0010] Crawford‐Williams, F. , Steen, M. , Esterman, A. , Fielder, A. & Mikocka‐Walus, A. (2015) ‘My midwife said that having a glass of red wine was actually better for the baby’: a focus group study of women and their partner’s knowledge and experiences relating to alcohol consumption in pregnancy. BMC Pregnancy and Childbirth, 15, 79. 25881173 10.1186/s12884-015-0506-3 PMC4389416

[acer14806-bib-0011] Day, N. , Helsel, A. , Sonon, K. & Goldschmidt, L. (2013) The association between prenatal alcohol exposure and behavior at 22 years of age. Alcoholism: Clinical and Experimental Research, 37, 1171–1178. 23442183 10.1111/acer.12073

[acer14806-bib-0012] Dejong, K. , Olyaei, A. & Lo, J.O. (2019) Alcohol use in pregnancy. Clinical Obstetrics and Gynecology, 62, 142–155. 30575614 10.1097/GRF.0000000000000414 PMC7061927

[acer14806-bib-0013] Del Boca, F. & Darkes, J. (2003) The validity of self‐reports of alcohol consumption: state of the science and challenges for research. Addiction, 98, 1–12. 10.1046/j.1359-6357.2003.00586.x 14984237

[acer14806-bib-0014] Denny, C. , Acero, C. , Naimi, T. & Kim, S. (2019) Consumption of alcohol beverages and binge drinking among pregnant women aged 18–44 years – United States, 2015–2017. MMWR. Morbidity and Mortality Weekly Report, 68, 365–368. 31022164 10.15585/mmwr.mm6816a1 PMC6483284

[acer14806-bib-0015] Dumas, A. , Toutain, S. & Simmat‐Durand, L. (2017) Alcohol use during pregnancy or breastfeeding: a national survey in France. Journal of Women’s Health, 26, 798–805. 10.1089/jwh.2016.6130 28281881

[acer14806-bib-0016] Ethen, M. , Ramadhani, T. , Scheuerle, A. , Canfield, M. , Wyszynski, D. , Druschel, C. et al. (2009) Alcohol consumption by women before and during pregnancy. Maternal and Child Health Journal, 13, 274–285. 18317893 10.1007/s10995-008-0328-2 PMC6090563

[acer14806-bib-0017] Finanger, T. , Spigset, O. , Gråwe, R.W. , Andreassen, T.N. , Løkken, T.N. , Aamo, T.O. et al. (2021) Phosphatidylethanol as blood biomarker of alcohol consumption in early pregnancy: an observational study in 4,067 pregnant women. Alcoholism: Clinical and Experimental Research, 45, 886–892. 33586791 10.1111/acer.14577

[acer14806-bib-0018] Graham, J. (2009) Missing data analysis: making it work in the real world. Annual Review of Psychology, 60, 549–576. 10.1146/annurev.psych.58.110405.085530 18652544

[acer14806-bib-0019] Grigsby, T. & Mclawhorn, J. (2019) Missing data techniques and the statistical conclusion validity of survey‐based alcohol and drug use research studies: a review and comment on reproducibility. Journal of Drug Issues, 49, 44–56.

[acer14806-bib-0020] Heino, A. & Gissler, M. (2020) Nordic perinatal statistics 2018: the proportion of parturients aged 35 and over is the highest in Finland of the Nordic countries. THL statistical report 13/2020.

[acer14806-bib-0021] Högberg, H. , Skagerström, J. , Spak, F. , Nilsen, P. & Larsson, M. (2016) Alcohol consumption among partners of pregnant women in Sweden: a cross sectional study. BMC Public Health, 16, 694. 27484750 10.1186/s12889-016-3338-9 PMC4971635

[acer14806-bib-0022] Hoyme, H. , Kalberg, W. , Elliott, A. , Blankenship, J. , Buckley, D. , Marais, A.‐S. et al. (2016) Updated clinical guidelines for diagnosing fetal alcohol spectrum disorders. Pediatrics, 138, e20154256. 27464676 10.1542/peds.2015-4256 PMC4960726

[acer14806-bib-0023] Huuskonen, P. , Keski‐Nisula, L. , Heinonen, S. , Voutilainen, S. , Tuomainen, T.P. , Pekkanen, J. et al. (2018) Kuopio birth cohort – design of a Finnish joint research effort for identification of environmental and lifestyle risk factors for the wellbeing of the mother and the newborn child. BMC Pregnancy and Childbirth, 18, 381. 30241516 10.1186/s12884-018-2013-9 PMC6150990

[acer14806-bib-0024] Jacobson, J. , Jacobson, S. , Sokol, R. & Ager, J. (1998) Relation of maternal age and pattern of pregnancy drinking to functionally significant cognitive deficit in infancy. Alcoholism: Clinical and Experimental Research, 22, 345–351. 9581639 10.1111/j.1530-0277.1998.tb03659.x

[acer14806-bib-0025] Jacobson, J. & Jacobson, S. (1999) Drinking moderately and pregnancy: effects on child development. Alcohol Research & Health, 23, 25–30. 10890795 PMC6761692

[acer14806-bib-0026] Jacobson, S. , Hoyme, H. , Carter, R. , Dodge, N. , Molteno, C. , Meintjes, E. et al. (2021) Evolution of the physical phenotype of fetal alcohol spectrum disorders from childhood through adolescence. Alcoholism: Clinical and Experimental Research, 45, 395–408. 33320363 10.1111/acer.14534

[acer14806-bib-0027] Jacobson, S. , Jacobson, J. , Sokol, R. , Martier, S. , Ager, J. & Kaplan, M. (1991) Maternal recall of alcohol, cocaine, and marijuana use during pregnancy. Neurotoxicology and Teratology, 13, 535–540. 1758408 10.1016/0892-0362(91)90062-2

[acer14806-bib-0028] Jones, K. , Smith, D.W. , Ulleland, C.N. & Streissguth, A.P. (1973) Pattern of malformation in offspring of chronic alcoholic mothers. Lancet, 1, 1267–1271. 4126070 10.1016/s0140-6736(73)91291-9

[acer14806-bib-0029] Jousilahti, P. , Salomaa, V. , Kuulasmaa, K. , Niemelä, M. & Vartiainen, E. (2005) Total and cause specific mortality among participants and non‐participants of population based health surveys: a comprehensive follow up of 54 372 Finnish men and women. Journal of Epidemiology and Community Health, 59, 310–315. 15767385 10.1136/jech.2004.024349 PMC1733044

[acer14806-bib-0030] Kautz‐Turnbull, C. , Petrenko, C. , Handley, E. , Coles, C. , Kable, J. , Wertelecki, W. et al. (2021) Partner influence as a factor in maternal alcohol consumption and depressive symptoms, and maternal effects on infant neurodevelopmental outcomes. Alcoholism: Clinical and Experimental Research, 45, 1265–1275. 33999430 10.1111/acer.14612 PMC8254755

[acer14806-bib-0031] Kitsantas, P. , Gaffney, K. , Wu, H. & Kastello, J. (2014) Determinants of alcohol cessation, reduction and no reduction during pregnancy. Archives of Gynecology and Obstetrics, 289, 771–779. 24150521 10.1007/s00404-013-3056-9

[acer14806-bib-0032] Kleinke, K. (2017) Multiple imputation under violated distributional assumptions: a systematic evaluation of the assumed robustness of predictive mean matching. Journal of Educational and Behavioral Statistics, 42, 371–404.

[acer14806-bib-0033] Mårdby, A.C. , Lupattelli, A. , Hensing, G. & Nordeng, H. (2017) Consumption of alcohol during pregnancy—a multinational European study. Women and Birth, 30, e207–213. 28111037 10.1016/j.wombi.2017.01.003

[acer14806-bib-0034] May, P.A. , Baete, A. , Russo, J. , Elliott, A.J. , Blankenship, J. , Kalberg, W.O. et al. (2014) Prevalence and characteristics of fetal alcohol spectrum disorders. Pediatrics, 134, 855–866. 25349310 10.1542/peds.2013-3319 PMC4210790

[acer14806-bib-0035] McCormack, C. , Hutchinson, D. , Burns, L. , Wilson, J. , Elliott, E. , Allsop, S. et al. (2017) Prenatal alcohol consumption between conception and recognition of pregnancy. Alcoholism: Clinical and Experimental Research, 41, 369–378. 28116821 10.1111/acer.13305

[acer14806-bib-0036] Mellingen, S. , Torsheim, T. & Thuen, F. (2013) Changes in alcohol use and relationship satisfaction in Norwegian couples during pregnancy. Substance Abuse: Treatment, Prevention, and Policy, 8, 5. 23356958 10.1186/1747-597X-8-5 PMC3565924

[acer14806-bib-0037] Morrow‐Tlucak, M. , Emhart, C. , Sokol, R. , Martier, S. & Ager, J. (1989) Underreporting of alcohol use in pregnancy: relationship to alcohol problem history. Alcoholism: Clinical and Experimental Research, 13, 399–401. 2665555 10.1111/j.1530-0277.1989.tb00343.x

[acer14806-bib-0038] Muggli, E. , Cook, B. , O’Leary, C. , Forster, D. & Halliday, J. (2015) Increasing accurate self‐report in surveys of pregnancy alcohol use. Midwifery, 31, e23–e28. 25467595 10.1016/j.midw.2014.11.003

[acer14806-bib-0039] Ní Shúilleabháin, A. , Barry, J. , Kelly, A. , O’Kelly, F. , Darker, C. & O’Dowd, T. (2013) Alcohol consumption in pregnancy: results from the general practice setting. Irish Journal of Medical Science, 183, 231–240. 23934378 10.1007/s11845-013-0996-9

[acer14806-bib-0040] Nilsen, P. , Holmqvist, M. , Hultgren, E. , Bendtsen, P. & Cedergren, M. (2008) Alcohol use before and during pregnancy and factors influencing change among Swedish women. Acta Obstetricia et Gynecologica Scandinavica, 87, 768–774. 18607824 10.1080/00016340802179830

[acer14806-bib-0041] Popova, S. , Lange, S. , Probst, C. , Gmel, G. & Rehm, J. (2017) Estimation of national, regional, and global prevalence of alcohol use during pregnancy and fetal alcohol syndrome: a systematic review and meta‐analysis. The Lancet Global Health, 5, e290–e299. 28089487 10.1016/S2214-109X(17)30021-9

[acer14806-bib-0042] Pryor, J. , Patrick, S.W. , Sundermann, A.C. , Wu, P. & Hartmann, K.E. (2017) Pregnancy intention and maternal alcohol consumption. Obstetrics and Gynecology, 129, 727–733. 28277356 10.1097/AOG.0000000000001933 PMC5679257

[acer14806-bib-0043] Reid, N. , Schölin, L. , Na Erng, M. , Montag, A. , Hanson, J. & Smith, L. (2021) Preconception interventions to reduce the risk of alcohol‐exposed pregnancies: a systematic review. Alcoholism: Clinical and Experimental Research, 45, 2414–2429. 34590331 10.1111/acer.14725

[acer14806-bib-0044] Roberts, S. , Wilsnack, S. , Foster, D. & Delucchi, K. (2014) Alcohol use before and during unwanted pregnancy. Alcoholism: Clinical and Experimental Research, 38, 2844–2852. 25336245 10.1111/acer.12544 PMC4245368

[acer14806-bib-0045] Rubin, D.B. (1987) Multiple imputation for nonresponse in surveys. New York, NY: Wiley.

[acer14806-bib-0046] Rubin, D.H. , Krasilnikoff, P. , Leventhal, J. , Berget, A. & Weile, B. (1988) Cigarette smoking and alcohol consumption during pregnancy by Danish women and their spouses – a potential source of fetal morbidity. American Journal of Drug and Alcohol Abuse, 14, 405–417. 3189260 10.3109/00952998809001560

[acer14806-bib-0054] Shawe, J. , Pate, D. , Joy, M. , Howden, B. , Barrett, G. & Stephenson, J. (2019) Preparation for fatherhood: A survey of men’s preconception health knowledge and behaviour in England. PLOS ONE, 14, e0213897. 10.1371/journal.pone.0213897 30893380 PMC6426231

[acer14806-bib-0047] Skagerstróm, J. , Chang, G. & Nilsen, P. (2011) Predictors of drinking during pregnancy: a systematic review. Journal of Women’s Health, 20, 901–913. 10.1089/jwh.2010.2216 PMC3159119 21671775

[acer14806-bib-0048] Streissguth, A. , Barr, H. & Sampson, P. (1990) Moderate prenatal alcohol exposure: effects on child IQ and learning problems at age 7 1/2 years. Alcoholism: Clinical and Experimental Research, 14, 662–669. 2264594 10.1111/j.1530-0277.1990.tb01224.x

[acer14806-bib-0049] Streissguth, A. , Sampson, P. , Olson, H. , Bookstein, F. , Barr, H. , Scott, M. et al. (1994) Maternal drinking during pregnancy: attention and short‐term memory in 14‐year‐old offspring—a longitudinal prospective study. Alcoholism: Clinical and Experimental Research, 18, 202–218. 8198221 10.1111/j.1530-0277.1994.tb00904.x

[acer14806-bib-0050] Van Ginkel, J. , Linting, M. , Rippe, R. & Van Der Voort, A. (2019) Rebutting existing misconceptions about multiple imputation as a method for handling missing data. Journal of Personality Assessment, 102, 297–308. 30657714 10.1080/00223891.2018.1530680

[acer14806-bib-0051] Waterson, E. , Evans, C. & Murray‐Lyon, I. (1990) Is pregnancy a time of changing drinking and smoking patterns for fathers as well as mothers? An initial investigation. British Journal of Addiction, 85, 389–396. 2334824 10.1111/j.1360-0443.1990.tb00655.x

[acer14806-bib-0052] van der Wulp, N. , Hoving, C. & de Vries, H. (2015) Partner’s influences and other correlates of prenatal alcohol use. Maternal and Child Health Journal, 19, 908–916. 25087003 10.1007/s10995-014-1592-y

[acer14806-bib-0053] Young‐Wolff, K. , Sarovar, V. , Alexeeff, S. , Adams, S. , Tucker, L. , Conway, A. et al. (2020) Trends and correlates of self‐reported alcohol and nicotine use among women before and during pregnancy, 2009–2017. Drug and Alcohol Dependence, 214, 108168. 32736316 10.1016/j.drugalcdep.2020.108168 PMC7423641

