# Supplementary material for: Self‐reported alcohol consumption of pregnant women and their partners correlates both before and during pregnancy: A cohort study with 21,472 singleton pregnancies
Source: Alcohol Clin Exp Res. 2022 May 15;46(5):797–808. doi: 10.1111/acer.14806 (PMC9321706; doi:10.1111/acer.14806)
Supplement: Supplementary file 1 — Fig S1 [file ACER-46-797-s007.pdf]

**Supporting Information**

Voutilainen et al.: Self-reported alcohol consumption of pregnant women and their partners correlates both before and during pregnancy: a cohort study with 21 472 singleton pregnancies

Alcoholism: Clinical and Experimental Research

| | | Frequency of drinking before pregnancy_Women [a] | Frequency of binge drinking before pregnancy_Women [a] | Average weekly alcohol dose before pregnancy_Women | AUDIT score before pregnancy_Women | Frequency of drinking during pregnancy_Women | Average weekly alcohol dose during pregnancy_Women | Age during pregnancy_Women | Frequency of drinking before pregnancy_Partners [a] | Frequency of binge drinking before pregnancy_Partners [a] | Average weekly alcohol dose before pregnancy_Partners | AUDIT score before pregnancy_Partners | Frequency of drinking during pregnancy_Partners | Average weekly alcohol dose during pregnancy_Partners | Age during pregnancy_Partners |
|---|---|---|---|---|---|---|---|---|---|---|---|---|---|---|---|
| Frequency of drinking before pregnancy_Women [a] | $r_s$ | | **0.51** | **0.75** | **0.69** | **0.12** | **0.11** | **0.05** | **0.63** | **0.44** | **0.53** | **0.46** | **0.49** | **0.48** | **0.04** |
| | N | 16 340 | 16 077 | 9 980 | 15 461 | 11 502 | 11 372 | 16 340 | 14 438 | 14 217 | 9 788 | 13 356 | 14 948 | 12 421 | 14 279 |
| Frequency of binge drinking before pregnancy_Women [a] | $r_s$ | **0.51** | | **0.69** | **0.85** | **0.09** | **0.11** | **-0.16** | **0.29** | **0.60** | **0.47** | **0.56** | **0.22** | **0.32** | **-0.14** |
| | N | 16 077 | 17 233 | 10 338 | 15 461 | 12 250 | 12 093 | 17 233 | 14 761 | 15 236 | 10 235 | 13 805 | 15 845 | 13 184 | 15 085 |
| Average weekly alcohol dose before pregnancy_Women | $r_s$ | **0.75** | **0.69** | | **0.84** | **0.22** | **0.19** | **-0.03** | **0.51** | **0.58** | **0.77** | **0.63** | **0.45** | **0.53** | -0.03 |
| | N | 9 980 | 10 338 | 12 343 | 9 610 | 7 959 | 7 839 | 12 343 | 9 224 | 9 435 | 9 270 | 8 617 | 10 897 | 8 827 | 10 517 |
| AUDIT score before pregnancy_Women | $r_s$ | **0.69** | **0.85** | **0.84** | | **0.11** | **0.15** | **-0.15** | **0.40** | **0.61** | **0.60** | **0.69** | **0.30** | **0.40** | **-0.14** |
| | N | 15 461 | 15 461 | 9 610 | 15 461 | 10 855 | 10 723 | 15 461 | 13 757 | 13 692 | 9 407 | 13 238 | 14 214 | 11 912 | 13 565 |
| Frequency of drinking during pregnancy_Women | $r_s$ | **0.12** | **0.09** | **0.22** | **0.11** | | **1.00** | **0.03** | **0.09** | **0.07** | **0.12** | **0.07** | **0.11** | **0.12** | **0.03** |
| | N | 11 502 | 12 250 | 7 959 | 10 855 | 14 301 | 13 919 | 14 301 | 10 653 | 10 950 | 7 025 | 9 797 | 12 532 | 9 810 | 11 922 |
| Average weekly alcohol dose during pregnancy_Women | $r_s$ | **0.11** | **0.11** | **0.19** | **0.15** | **1.00** | | -0.01 | **0.06** | **0.08** | **0.11** | **0.09** | **0.07** | **0.11** | 0.00 |
| | N | 11 372 | 12 093 | 7 839 | 10 723 | 13 919 | 14 101 | 14 101 | 10 519 | 10 798 | 6 893 | 9 669 | 12 309 | 9 721 | 11 756 |
| Age during pregnancy_Women | $r_s$ | **0.05** | **-0.16** | **-0.03** | **-0.15** | **0.03** | -0.01 | | **0.08** | **-0.12** | **-0.03** | **-0.13** | **0.09** | **0.04** | **0.75** |
| | N | 16 340 | 17 233 | 12 343 | 15 461 | 14 301 | 14 101 | 21 472 | 15 162 | 15 515 | 11 089 | 14 010 | 17 371 | 13 933 | 17 535 |
| Frequency of drinking before pregnancy_Partners [a] | $r_s$ | **0.63** | **0.29** | **0.51** | **0.40** | **0.09** | **0.06** | **0.08** | | **0.53** | **0.65** | **0.60** | **0.81** | **0.73** | **0.09** |
| | N | 14 438 | 14 761 | 9 224 | 13 757 | 10 653 | 10 519 | 15 162 | 15 162 | 14 781 | 9 864 | 14 010 | 14 950 | 12 802 | 13 561 |
| Frequency of binge drinking before pregnancy_Partners [a] | $r_s$ | **0.44** | **0.60** | **0.58** | **0.61** | **0.07** | **0.08** | **-0.12** | **0.53** | | **0.72** | **0.84** | **0.48** | **0.60** | **-0.10** |
| | N | 14 217 | 15 236 | 9 435 | 13 692 | 10 950 | 10 798 | 15 515 | 14 781 | 15 515 | 10 000 | 14 010 | 15 322 | 13 235 | 13 907 |
| Average weekly alcohol dose before pregnancy_Partners | $r_s$ | **0.53** | **0.47** | **0.77** | **0.60** | **0.12** | **0.11** | **-0.03** | **0.65** | **0.72** | | **0.80** | **0.65** | **0.73** | -0.01 |
| | N | 9 788 | 10 235 | 9 270 | 9 407 | 7 025 | 6 893 | 11 089 | 9 864 | 10 000 | 11 089 | 9 358 | 10 973 | 9 561 | 9 943 |
| AUDIT score before pregnancy_Partners | $r_s$ | **0.46** | **0.56** | **0.63** | **0.69** | **0.07** | **0.09** | **-0.13** | **0.60** | **0.84** | **0.80** | | **0.51** | **0.64** | **-0.09** |
| | N | 13 356 | 13 805 | 8 617 | 13 238 | 9 797 | 9 669 | 14 010 | 14 010 | 14 010 | 9 358 | 14 010 | 13 861 | 12 056 | 12 596 |
| Frequency of drinking during pregnancy_Partners | $r_s$ | **0.49** | **0.22** | **0.45** | **0.30** | **0.11** | **0.07** | **0.09** | **0.81** | **0.48** | **0.65** | **0.51** | | **0.91** | **0.10** |
| | N | 14 948 | 15 845 | 10 897 | 14 214 | 12 532 | 12 309 | 17 371 | 14 950 | 15 322 | 10 973 | 13 861 | 17 371 | 13 755 | 15 401 |
| Average weekly alcohol dose during pregnancy_Partners | $r_s$ | **0.48** | **0.32** | **0.53** | **0.40** | **0.12** | **0.11** | **0.04** | **0.73** | **0.60** | **0.73** | **0.64** | **0.91** | | **0.05** |
| | N | 12 421 | 13 184 | 8 827 | 11 912 | 9 810 | 97 21 | 13 933 | 12 802 | 13 235 | 9 561 | 12 056 | 13 755 | 13 933 | 12 445 |
| Age during pregnancy_Partners | $r_s$ | **0.04** | **-0.14** | -0.03 | **-0.14** | **0.03** | 0.00 | **0.75** | **0.09** | **-0.10** | -0.01 | **-0.09** | **0.10** | **0.05** | |
| | N | 14 279 | 15 085 | 10 517 | 13 565 | 11 922 | 11 756 | 17 535 | 13 561 | 13 907 | 9 943 | 12 596 | 15 401 | 12 445 | 17 535 |

**Scale:** 0.60, 0.50, 0.40, 0.30, 0.20, 0.10, 0.00, -0.10, -0.20

**Figure S1. Correlation heatmap of the self-reported alcohol use and the age variables of the women and their partners in the original data.** Statistically significant correlations ($p < 0.0005$) are indicated with bold. The valid number of cases (**n**) in each correlation is indicated below the Spearman rho's correlation coefficient (**$r_s$**). N = 21 472. The grey rectangles indicate correlations where the correlation was statistically significant in the original data but not in the multiple imputed data, and the green rectangles indicate correlations where the correlation was statistically significant in the multiple imputed data but not in the original data. Black font in the correlation coefficients indicates that the difference between the original and the multiple imputed correlation coefficients differ more or equal to 0.15, while the blue indicates a difference smaller than that. [a] The before pregnancy frequency of drinking and frequency of binge drinking are questions in the AUDIT questionnaire.
