# Supplementary material for: Self‐reported alcohol consumption of pregnant women and their partners correlates both before and during pregnancy: A cohort study with 21,472 singleton pregnancies
Source: Alcohol Clin Exp Res. 2022 May 15;46(5):797–808. doi: 10.1111/acer.14806 (PMC9321706; doi:10.1111/acer.14806)
Supplement: Supplementary file 2 — Fig S2 [file ACER-46-797-s004.pdf]

Supporting Information

Voutilainen et al.: Self-reported alcohol consumption of pregnant women and their partners correlates both before and during pregnancy: a cohort study with 21 472 singleton pregnancies

Alcoholism: Clinical and Experimental Research

	Frequency of drinking before pregnancy_Women [a]	Frequency of binge drinking before pregnancy_Women [a]	Average weekly alcohol dose before pregnancy_Women	AUDIT score before pregnancy_Women	Frequency of drinking during pregnancy_Women	Average weekly alcohol dose during pregnancy_Women	Age during pregnancy_Women	Frequency of drinking before pregnancy_Partners [a]	Frequency of binge drinking before pregnancy_Partners [a]	Average weekly alcohol dose before pregnancy_Partners	AUDIT score before pregnancy_Partners	Frequency of drinking during pregnancy_Partners	Average weekly alcohol dose during pregnancy_Partners	Age during pregnancy_Partners
Frequency of drinking before pregnancy_Women [a]		**0.47**	**0.36**	**0.67**	**0.28**	**0.28**	0.03	**0.48**	**0.38**	**0.27**	**0.45**	**0.28**	**0.23**	0.03
Frequency of binge drinking before pregnancy_Women [a]	**0.47**		**0.32**	**0.78**	**0.22**	**0.23**	**-0.11**	**0.30**	**0.51**	**0.24**	**0.54**	0.13	0.16	**-0.09**
Average weekly alcohol dose before pregnancy_Women	**0.36**	**0.32**		**0.36**	**0.39**	**0.37**	**-0.06**	**0.20**	**0.22**	**0.56**	**0.21**	**0.24**	**0.32**	**-0.05**
AUDIT score before pregnancy_Women	**0.67**	**0.78**	**0.36**		**0.24**	**0.26**	**-0.09**	**0.40**	**0.56**	**0.28**	**0.69**	0.16	0.18	**-0.08**
Frequency of drinking during pregnancy_Women	**0.28**	**0.22**	**0.39**	**0.24**		0.81	0.00	**0.18**	0.15	**0.22**	0.15	**0.20**	**0.19**	0.01
Average weekly alcohol dose during pregnancy_Women	**0.28**	**0.23**	**0.37**	**0.26**	0.81		0.00	**0.18**	**0.16**	**0.20**	**0.17**	**0.18**	**0.18**	0.00
Age during pregnancy_Women	0.03	**-0.11**	**-0.06**	**-0.09**	0.00	0.00		**0.05**	**-0.06**	**-0.04**	**-0.06**	**0.06**	0.00	**0.75**
Frequency of drinking before pregnancy_Partners [a]	**0.48**	**0.30**	**0.20**	**0.40**	**0.18**	**0.18**	**0.05**		**0.53**	**0.40**	**0.62**	**0.48**	**0.34**	**0.06**
Frequency of binge drinking before pregnancy_Partners [a]	**0.38**	**0.51**	**0.22**	**0.56**	0.15	**0.16**	**-0.06**	**0.53**		**0.38**	**0.81**	**0.26**	0.27	**-0.04**
Average weekly alcohol dose before pregnancy_Partners	**0.27**	**0.24**	**0.56**	**0.28**	**0.22**	**0.20**	**-0.04**	**0.40**	**0.38**		**0.38**	**0.49**	**0.53**	-0.01
AUDIT score before pregnancy_Partners	**0.45**	**0.54**	**0.21**	**0.69**	0.15	**0.17**	**-0.06**	**0.62**	**0.81**	**0.38**		0.27	**0.29**	**-0.03**
Frequency of drinking during pregnancy_Partners	**0.28**	0.13	**0.24**	0.16	**0.20**	**0.18**	**0.06**	**0.48**	**0.26**	**0.49**	0.27		**0.73**	**0.07**
Average weekly alcohol dose during pregnancy_Partners	**0.23**	0.16	**0.32**	0.18	**0.19**	**0.18**	0.00	**0.34**	0.27	**0.53**	**0.29**	**0.73**		0.02
Age during pregnancy_Partners	0.03	**-0.09**	**-0.05**	**-0.08**	0.01	0.00	**0.75**	**0.06**	**-0.04**	-0.01	**-0.03**	**0.07**	0.02	

Scale:

0.60
0.50
0.40
0.30
0.20
0.10
0.00
-0.10
-0.20

Figure S2. Sensitivity analysis of the correlation coefficients. The Spearman rho's correlation coefficients of alcohol use and age variables of the women and their partners are those of the total score level multiple imputed data (MI = 40). Thus, the N in each cell is 21 472. Statistically significant correlations ($p < 0.0005$) are indicated with bold. There were no differences in the statistical significance of the correlation coefficients between the two multiple imputations. The differences in the coefficients are indicated with blue font. [a] The before pregnancy frequency of drinking and frequency of binge drinking are both questions in the AUDIT questionnaire, which explains the stronger correlation between them and the total AUDIT score than the rest of the variables.
