# Supplementary material for: Self‐reported alcohol consumption of pregnant women and their partners correlates both before and during pregnancy: A cohort study with 21,472 singleton pregnancies
Source: Alcohol Clin Exp Res. 2022 May 15;46(5):797–808. doi: 10.1111/acer.14806 (PMC9321706; doi:10.1111/acer.14806)
Supplement: Supplementary file 3 — Fig S3 [file ACER-46-797-s005.pdf]

Supporting Information
Voutilainen et al.: Self-reported alcohol consumption of pregnant women and their partners correlates both before and during pregnancy: a cohort study with 21 472 singleton pregnancies.
Alcoholism: Clinical and Experimental Research

Randomly selected one pregnancy sample

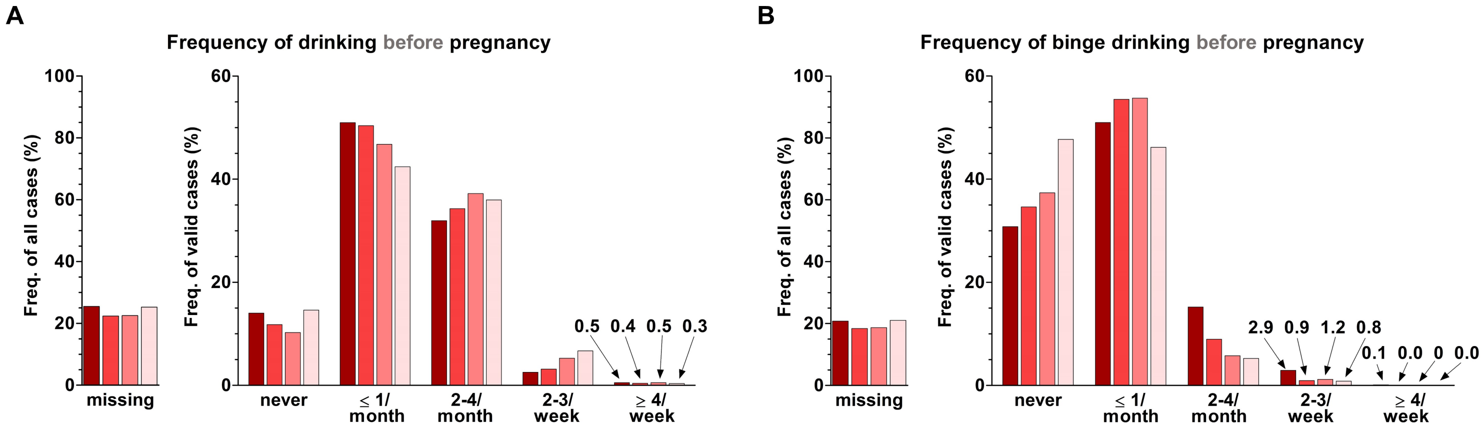

Reported	using alcohol before pregnancy	regular binge drinking before pregnancy
Independent sample (% of women)	87.4	9.9
Original Cohort (% of pregnancies)	85.7	9.2

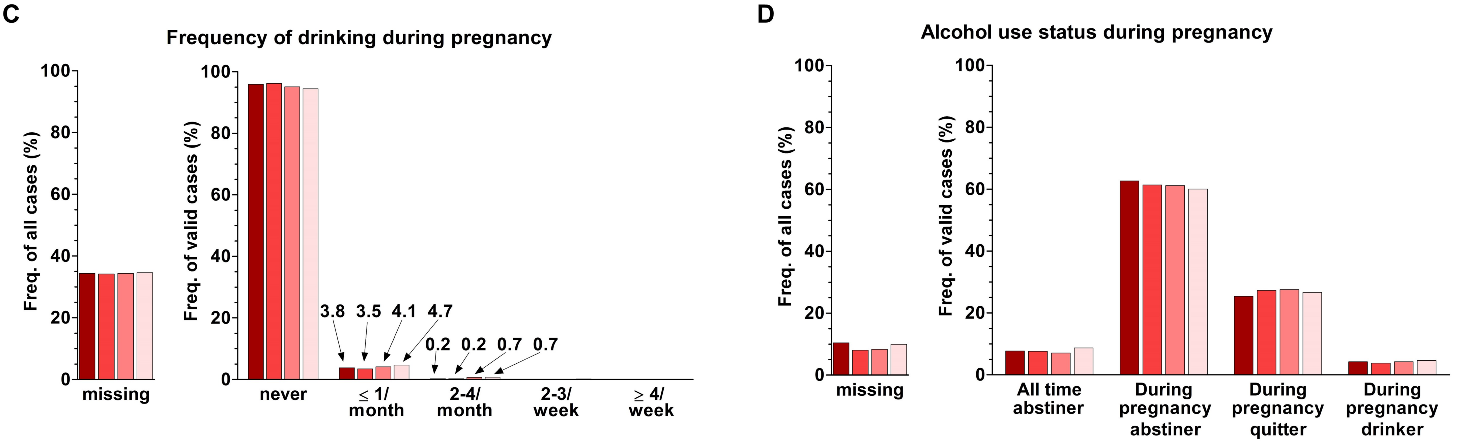

Reported	continuing alcohol use during pregnancy	quitting alcohol use during pregnancy	Fetal Alcohol Exposure
Independent sample (% of women)	4.6	26.8	31.0
Original Cohort (% of pregnancies)	4.4	25.7	29.8

Figure S3. Sensitivity analysis for the independent cases frequency data. N = 14 822 women. For independent case analysis one pregnancy was randomly selected for each woman in the cohort. The bar graphs represent the women's **A** frequency of drinking before pregnancy (n of valid cases i.e., the number of women having answered to the question was 11 292); **B** frequency of binge drinking before pregnancy (n of valid cases 11 906); **C** frequency of drinking during pregnancy (n of valid cases 9729); and **D** alcohol use status during pregnancy (n of valid cases 13 472). Note that multiple imputation (MI) cannot be used to report frequencies. Thus, the frequency of all cases bar graphs show the proportion of women in each age group having a missing answer in the original data and the frequency of valid cases bar graphs illustrates the distribution of the valid answers in each age group (each age group gives a total of 100%) in the original data. Note, that the most important general comparisons stated under these graphs were similar in both the independent sample and nonindependent cohort (n = 21 472 pregnancies). This indicates that the results of our nonindependent pregnancies (i.e. proportion of pregnancies) can be generalized to proportion of women in our cohort. For example, in **A** in the independent sample in total 87.4% of women reported using alcohol before pregnancy whereas in the whole cohort women did so in 85.7% of the pregnancies. Compare panels **A** & **B** to **Fig. 3 A & C**, respectively, and panels **C** & **D** to **Fig. 4 A & C**, respectively.
