# Supplementary material for: Self‐reported alcohol consumption of pregnant women and their partners correlates both before and during pregnancy: A cohort study with 21,472 singleton pregnancies
Source: Alcohol Clin Exp Res. 2022 May 15;46(5):797–808. doi: 10.1111/acer.14806 (PMC9321706; doi:10.1111/acer.14806)
Supplement: Supplementary file 4 — Fig S4 [file ACER-46-797-s002.pdf]

Supporting Information

Voutilainen et al.: Self-reported alcohol consumption of pregnant women and their partners correlates both before and during pregnancy: a cohort study with 21 472 singleton pregnancies

Alcoholism: Clinical and Experimental Research

		Frequency of drinking before pregnancy_Mother [a]	Frequency of binge drinking before pregnancy_Mother [a]	Average weekly alcohol dose before pregnancy_Mother	AUDIT score before pregnancy_Mother	Frequency of drinking during pregnancy_Mother	Average weekly alcohol dose during pregnancy_Mother	Age during pregnancy_Mother	Frequency of drinking before pregnancy_Partner [a]	Frequency of binge drinking before pregnancy_Partner [a]	Average weekly alcohol dose before pregnancy_Partner	AUDIT score before pregnancy_Partner	Frequency of drinking during pregnancy_Partner	Average weekly alcohol dose during pregnancy_Partner	Age during pregnancy_Partner
Frequency of drinking [a] before pregnancy_Mother	r_s		**0.50**	**0.72**	**0.67**	**0.12**	**0.10**	**0.06**	**0.62**	**0.43**	**0.50**	**0.44**	**0.47**	**0.46**	**0.05**
	N	11 292	11 097	6881	10 673	7818	7719	11 292	9906	9754	6720	9149	10 262	8532	9927
Frequency of binge drinking [a] before pregnancy_Mother	r_s	**0.50**		**0.68**	**0.84**	**0.09**	**0.11**	**-0.16**	**0.27**	**0.59**	**0.46**	**0.55**	**0.19**	**0.29**	**-0.14**
	N	11 097	11 906	7133	10 673	8328	8208	11 906	10 121	10 462	7025	9457	10 882	9060	10 481
Average weekly alcohol dose before pregnancy_Mother	r_s	**0.72**	**0.68**		**0.82**	**0.21**	**0.19**	-0.04	**0.47**	**0.56**	**0.75**	**0.60**	**0.42**	**0.49**	-0.03
	N	6881	7133	8498	6619	5345	5257	8498	6331	6491	6343	5914	7465	6062	7283
AUDIT score before pregnancy_Mother	r_s	**0.67**	**0.84**	**0.82**		**0.10**	**0.14**	**-0.15**	**0.37**	**0.60**	**0.58**	**0.68**	**0.27**	**0.37**	**-0.13**
	N	10 673	10 673	6619	10 673	7363	7261	10 673	9438	9399	6452	9064	9760	8171	9414
Frequency of drinking during pregnancy_Mother	r_s	**0.12**	**0.09**	**0.21**	**0.10**		**1.00**	0.03	**0.09**	**0.07**	**0.11**	**0.06**	**0.11**	**0.12**	**0.04**
	N	7818	8328	5345	7363	9729	9451	9729	7195	7397	4673	6588	8442	6588	8168
Average weekly alcohol dose during pregnancy_Mother	r_s	**0.10**	**0.11**	**0.19**	**0.14**	**1.00**		-0.02	**0.06**	**0.08**	**0.11**	**0.09**	**0.07**	**0.11**	0.00
	N	7719	8208	5257	7261	9451	9575	9575	7091	7284	4578	6495	8276	6525	8035
Age during pregnancy_Mother	r_s	**0.06**	**-0.16**	-0.04	**-0.15**	0.03	-0.02		**0.09**	**-0.13**	-0.03	**-0.13**	**0.09**	**0.04**	**0.75**
	N	11 292	11 906	8498	10 673	9729	9575	14 822	10 411	10 654	7582	9599	11 898	9552	12 201
Frequency of drinking [a] before pregnancy_Partner	r_s	**0.62**	**0.27**	**0.47**	**0.37**	**0.09**	**0.06**	**0.09**		**0.51**	**0.63**	**0.58**	**0.80**	**0.72**	**0.10**
	N	9906	10 121	6331	9438	7195	7091	10 411	10 411	10 133	6768	9599	10 253	8784	9406
Frequency of binge drinking [a] before pregnancy_Partner	r_s	**0.43**	**0.59**	**0.56**	**0.60**	**0.07**	**0.08**	**-0.13**	**0.51**		**0.72**	**0.84**	**0.46**	**0.58**	**-0.10**
	N	9754	10 462	6491	9399	7397	7284	10 654	10 133	10 654	6869	9599	10 512	9099	9645
Average weekly alcohol dose before pregnancy_Partner	r_s	**0.50**	**0.46**	**0.75**	**0.58**	**0.11**	**0.11**	-0.03	**0.63**	**0.72**		**0.79**	**0.62**	**0.71**	0.00
	N	6720	7025	6343	6452	4673	4578	7582	6768	6869	7582	6417	7503	6550	6858
AUDIT score before pregnancy_Partner	r_s	**0.44**	**0.55**	**0.60**	**0.68**	**0.06**	**0.09**	**-0.13**	**0.58**	**0.84**	**0.79**		**0.48**	**0.62**	**-0.09**
	N	9149	9457	5914	9064	6588	6495	9599	9599	9599	6417	9599	9489	8250	8722
Frequency of drinking during pregnancy_Partner	r_s	**0.47**	**0.19**	**0.42**	**0.27**	**0.11**	**0.07**	**0.09**	**0.80**	**0.46**	**0.62**	**0.48**		**0.90**	**0.11**
	N	10 262	10 882	7465	9760	8442	8276	11 898	10 253	10 512	7503	9489	11 898	9417	10 647
Average weekly alcohol dose during pregnancy_Partner	r_s	**0.46**	**0.29**	**0.49**	**0.37**	**0.12**	**0.11**	**0.04**	**0.72**	**0.58**	**0.71**	**0.62**	**0.90**		**0.05**
	N	8532	9060	6062	8171	6588	6525	9552	8784	9099	6550	8250	9417	9552	8605
Age during pregnancy_Partner	r_s	**0.05**	**-0.14**	-0.03	**-0.13**	**0.04**	0.00	**0.75**	**0.10**	**-0.10**	0.00	**-0.09**	**0.11**	**0.05**	
	N	9927	10481	7283	9414	8168	8035	12 201	9406	9645	6858	8722	10 647	8605	12 201

Scale: 0.60, 0.50, 0.40, 0.30, 0.20, 0.10, 0.00, -0.10, -0.20

Figure S4. Sensitivity analysis for the independent cases correlation coefficient. N = 14 822 women. For independent case analysis one pregnancy for each woman in the cohort was randomly selected. These coefficients were compared to those obtained from analyzing all the pregnancies in the cohort (n = 21 472 pregnancies) (see **Figure S1.**). Coefficients in these two analyses were similar (i.e., the differences were less than 0.05), there were no differences in their direction, and no major differences in their statistical significances. Thus, although our cohort deviated from the assumption of independence, this has very little effect on results and did not affect the interpretation of the results. Statistically significant correlations ($p < 0.0005$) are indicated in bold. The valid number of cases (n) in each correlation is indicated below the Spearman rho's correlation coefficient (r_s). [a] The before-pregnancy frequency of drinking and the frequency of binge drinking are questions in the AUDIT questionnaire.
