# Supplementary material for: Self‐reported alcohol consumption of pregnant women and their partners correlates both before and during pregnancy: A cohort study with 21,472 singleton pregnancies
Source: Alcohol Clin Exp Res. 2022 May 15;46(5):797–808. doi: 10.1111/acer.14806 (PMC9321706; doi:10.1111/acer.14806)
Supplement: Supplementary file 5 — Table S1 [file ACER-46-797-s001.pdf]

**Supporting Information**
Voutilainen et al.
Alcoholism: Clinical and Experimental Research

**Table S1. The mean and standard deviations of the variables in the original data.** Red **bold** font indicates where the estimates of the original data differ from those obtained from item score level multiple imputed estimates.

| **Birth outcomes** | **n** | **(%)** | **Mean** | **±** | **SD** | | | | | |
|---|---|---|---|---|---|---|---|---|---|---|
| **Birthweight (g)** | 21 464 | 100.0 | 3477.**7** | ± | 57**6.9** | | | | | |
| **Head circumference (cm)** | 20 668 | 95.8 | 3**5.0** | ± | **1.9** | | | | | |
| **Umbilical cord length (cm)** | 21 239 | 98.9 | 59.**8** | ± | 13.8 | | | | | |
| **Post-membrane weight (g)** | 21 179 | 98.6 | 598.**2** | ± | 131.**5** | | | | | |
| | **Women** | | | | | **Partners** | | | | |
| **Characteristics of the parents** | **n** | **(%)** | **Mean** | **±** | **SD** | **n** | **(%)** | **Mean** | **±** | **SD** |
| **Age (years during pregnancy)** [a] | - | - | - | | - | 17 535 | 81.6 | 31.**4** | ± | 6.**3** |
| **BMI before pregnancy** [b] | 20 493 | 95.4 | 24.**9** | ± | 5.**2** | - | - | - | | - |
| **AUDIT score before pregnancy** | 15 461 | 72.0 | **3.0** | ± | **2.6** | 14 010 | 65.3 | **4.6** | ± | **3.4** |
| **Self-reported weekly alcohol dose** [c] | | | | | | | | | | |
| **Before pregnancy** | | | | | | | | | | |
| **All** | 12 343 | 57.5 | 1.8 | ± | 2.**4** | 11 089 | 51.6 | 3.**4** | ± | **4.0** |
| **Dose ≥ 1** | 8 462 | 39.4 | 2.**6** | ± | 2.**6** | 9 217 | 42.9 | **4.1** | ± | **4.0** |
| **Self-reported weekly alcohol dose** [c] | | | | | | | | | | |
| **During pregnancy** | | | | | | | | | | |
| **All** | 14 101 | 65.7 | **0.1** | ± | **0.9** | 13 933 | 64.9 | 3.**6** | ± | 4.**9** |
| **Dose ≥ 1** | 433 | 2.0 | 3.**0** | ± | **3.9** | 11 177 | 52.1 | 4.**5** | ± | **5.1** |

[a] The age of all the mothers was known; [b] Neither weight nor height of the partners was collected; [c] One dose of alcohol equals 12 g of pure ethanol.
