# Supplementary material for: Self‐reported alcohol consumption of pregnant women and their partners correlates both before and during pregnancy: A cohort study with 21,472 singleton pregnancies
Source: Alcohol Clin Exp Res. 2022 May 15;46(5):797–808. doi: 10.1111/acer.14806 (PMC9321706; doi:10.1111/acer.14806)
Supplement: Supplementary file 6 — Table S2 [file ACER-46-797-s008.pdf]

Supporting Information
Voutilainen et al.
Alcoholism: Clinical and Experimental Research

Table S2. Sensitivity analysis of the multiple imputed estimates. Differences between the mean and standard deviations of the item and total score level multiple imputed variables are highlighted in red. Due to great similarity of the estimates, no age division was made for women and their partners.

	**Multiple Imputation Characteristics**	**Valid n[a]**	**% of all cases**	**Item score level Mean**	±	**SD**	**Total score level Mean**	±	**SD**
**Birth outcomes**	**Birthweight (g)**	21 464	99.96	3 477.61	±	576.96	3477.61	±	576.96
	**Head circumference (cm)**	20 668	96.26	34.90	±	2.18	34.90	±	2.18
	**Umbilical cord length (cm)**	21 239	98.91	59.74	±	13.78	59.75	±	13.78
	**Post-membrane weight (g)**	21 179	98.64	597.99	±	131.70	597.99	±	131.70
**Women**	**BMI before pregnancy**	20 493	95.44	24.82	±	5.09	24.82	±	5.09
	**AUDIT score before pregnancy**	15 461	72.01	7.16	±	8.79	7.06	±	8.57
	**Self-reported weekly alcohol dose [b]**								
	**Before pregnancy**								
	**All**	12 343	57.48	1.81	±	2.31	1.81	±	2.31
	**Dose ≥ 1**	8462	39.41	2.48	±	2.37	2.48	±	2.37
	**During pregnancy**								
	**All**	14 101	65.67	0.97	±	2.11	0.97	±	2.11
	**Dose ≥ 1**	433	2.02	3.56	±	2.67	3.56	±	2.67
**Partners**	**Age (years during pregnancy)**	17 535	81.66	31.47	±	6.36	31.47	±	6.36
	**AUDIT score before pregnancy**	14 010	65.25	9.88	±	9.17	9.80	±	9.00
	**Self-reported weekly alcohol dose [b]**								
	**Before pregnancy**								
	**All**	11 089	51.64	3.31	±	3.69	3.31	±	3.69
	**Dose ≥ 1**	9217	42.93	3.91	±	3.71	3.91	±	3.71
	**During pregnancy**								
	**All**	13 933	64.89	3.09	±	4.55	3.09	±	4.55
	**Dose ≥ 1**	11 177	52.05	4.35	±	4.86	4.35	±	4.86

[a] the number of cases with a valid answer in the original data; [b] One dose of alcohol equals 12 g of pure ethanol. Abbreviations: BMI = body mass index; MI = multiple imputation.
