# Supplementary material for: Self‐reported alcohol consumption of pregnant women and their partners correlates both before and during pregnancy: A cohort study with 21,472 singleton pregnancies
Source: Alcohol Clin Exp Res. 2022 May 15;46(5):797–808. doi: 10.1111/acer.14806 (PMC9321706; doi:10.1111/acer.14806)
Supplement: Supplementary file 7 — Table S3 [file ACER-46-797-s006.pdf]

Supporting Information
Voutilainen et al.
Alcoholism: Clinical and Experimental Research

Table S3. Sensitivity analysis for independent cases. N = 14 822 women. For independent case analysis one pregnancy was randomly selected for each woman in the cohort. **Red bold font** indicates the level where the estimates of the randomly selected one pregnancy per women sample (n = 14 822) differed from results obtained from data including all 21 472 pregnancies.

**Birth outcomes**	**n**	**(%)**	**Mean**	±	**SD**		
**Duration of pregnancy (days)**	14 822	100	276.**7**	±	1**5.2**		
**Birthweight (g)**	14 815	100.0	34**56.2**	±	5**99.2**		
**Head circumference (cm)**	14 148	95.5	34.**9**	±	2.**0**		
**Umbilical cord length (cm)**	14 648	98.8	59.**8**	±	13.8		
**Post-membrane weight (g)**	14 609	98.6	59**6.3**	±	13**4.4**		
**Characteristics of the women**	**n**	**(%)**	**Mean**	**±**	**SD**	**Diff [a] (%)**	**Diff [b] (pp)**
**Age (years during pregnancy)**	14 822	100	30.0		5.5	+0.7	
	3168	21.4					-0.7
	3958	26.7					-1.0
	3794	25.6					-0.6
	3902	26.3					+2.3
**Gravidity**	14 822	100.0	2.5	±	1.8	-3.8	
**Parity**	14 822	100.0	1.**0**	±	1.**3**	-9.1	
	6832	46.1					+4.0
	4447	30.0					-1.8
	3543	23.9					-2.2
**Used ART**	753	5.1					+0.4
**Marital status (at childbirth)**	14 703	99.2					-0.2
In a relationship [c]	11 968	80.7					-1.1
Single [d]	14 703	18.5					+1.1
**BMI before pregnancy**	14 141	95.4	24.9	±	5.2	+0.4	
**AUDIT score before pregnancy**	15 461	72.0	3.**1**	±	2.6	+3.3	
**Self-reported weekly alcohol dose [e]**							
**Before pregnancy**							
**All**	12 343	57.5	1.**9**	±	2.**6**	+5.6	
**Dose ≥ 1**	6039	39.4	2.**7**	±	2.**7**	+3.8	
**During pregnancy**							
**All**	14 101	65.7	0.1	±	1.**0**	0	
**Dose ≥ 1**	303	2.0	3.**2**	±	**4.4**	+6.7	

[a] Difference in mean between the random pregnancy sample and the total cohort; [b] pp = Percentage point differences in the frequencies between the random pregnancy sample and the total cohort; [c] Married, engaged, or living with a partner; [e] single, widowed, or not living with a partner; [e] One dose of alcohol equals 12 g of pure ethanol.
