# Supplementary material for: Self‐reported alcohol consumption of pregnant women and their partners correlates both before and during pregnancy: A cohort study with 21,472 singleton pregnancies
Source: Alcohol Clin Exp Res. 2022 May 15;46(5):797–808. doi: 10.1111/acer.14806 (PMC9321706; doi:10.1111/acer.14806)
Supplement: Supplementary file 8 — Method S1 [file ACER-46-797-s003.pdf]

Supporting Methods

Paper title: Self-reported alcohol consumption of pregnant women and their partners correlates both before and during pregnancy: a cohort study with 21 472 singleton pregnancies

Authors: Voutilainen Taija; Rysä Jaana; Keski-Nisula Leea; Kärkkäinen Olli

Multiple Imputation (MI)

The missing data was accounted for by multiple imputation (MI) using IBM SPSS Statistics Software (v. 27.0.1.0). The missingness in variables varied from 0% to as high as 48.4% in partners' average weekly alcohol doses before pregnancy (**Table SM1**). Although 14.2% of the data values were missing, only about 8.9% of the 21 472 pregnancy cases were complete.

Because the Little's Missing Completely At Random (MCAR) Test showed that the missing values were not MCAR ($p < 0.0001$), running a complete case analysis (CCA) would have both significantly decreased the statistical power and increased the probability of biased estimates. (Graham, 2009; De Silva et al., 2019) The analysis of the missing pattern revealed the missingness to be nonmonotonic. (Harel et al., 2018) Therefore, a Multiple Imputation by Chained Equation (MICE) i.e. Fully Conditional Specification (FCS) utilizing the Markov Chain Monte Carlo (MCMC) algorithm to impute the missing data was used. To be specific, because many of the variables were non-normally distributed and heavily skewed, (Van Ginkel et al., 2019) we used MI with a predictive mean matching (PMM) method ($M = 40$, $k = 5$). PMM applies regression analysis to find a pool of cases (k-nearest values, here $k = 5$) (Kleinke, 2017) that closely resemble a case with a missing value. It then randomly selects one case whose value it imputes in the place of the missing value. Thus, values outside the range of observed values cannot occur and the nonlinear relations are maintained more intact.

The Alcohol Use Disorders Identification Test (AUDIT) is a multi-item instrument composed of ten questions. If an individual has a missing value in one or more of the questions, then also the total score will be missing. In our cohort, the total AUDIT score of the women and/or their partners was missing in about one third of the 21 472 pregnancies. Of those with a missing total AUDIT score, one third of women and one fourth of partners had a missing value in a range

from one to seven AUDIT questions, while in the remainder, the answers to eight or more questions were missing. Therefore, the main imputation was done in the item score level, (Eekhout et al., 2014) where each missing AUDIT question was first imputed and then the total scores were calculated. However, because the number of missing questions per individual can affect the results, we also performed a sensitivity analysis by comparing the results of the item score level MI to the results of a total score level MI. In the total score level MI, the total AUDIT score was directly imputed. Both of the MI models included 75 predictor variables, of which 49 were imputed in the item score level and 35 in the total score level imputation. The specifications of the two MI models are presented in **Table SM1**.

In both MIs, the number of imputations was 40 (M = 40), because the fraction of missing information in some variables was over 0.90, (Graham, Olchowski and Gilreath, 2007) and doing more imputations in this large-scale epidemiological data would not have been feasible. The number of maximum iterations was set to twenty and the convergence of the scale level variables was evaluated after both MIs. (Karangwa, Kotze and Blignaut, 2016; Grigsby and Mclawhorn, 2019) Here is an example of the SPSS Syntax for the MI:

```
SET RNG=MT MTINDEX=Same number in both MIs.
DATASET DECLARE IMPUTED_SPSS_date.
DATASET DECLARE ITERATION_HISTORY_date.
MULTIPLE IMPUTATION List of variables in the model.
  /IMPUTE METHOD=FCS MAXITER=20 NIMPUTATIONS=40
SCALELEMODEL=PMM(5) INTERACTIONS=NONE
  SINGULAR=1E-007 MAXPCTMISSING=NONE MAXCASEDRAWS=300
MAXPARAMDRAWS=10 MAXMODELPARAM=5000
  /CONSTRAINTS Variable_1 ( ROLE=IND) *
  /CONSTRAINTS Variable_2 ( ROLE=IND) *
  /MISSINGSUMMARIES NONE
  /IMPUTATIONSUMMARIES MODELS DESCRIPTIVES
  /OUTFILE IMPUTATIONS=IMPUTED_SPSS_date
FCSITERATIONS=ITERATION_HISTORY_date.
EXECUTE.
```

*** All the variables that are not imputed but used as predictors only listed in Constraints.**

Supporting Methods

The formulas used to calculate the reported multiple imputed results are listed below:

The pooled estimate $\overline{Q}_m$ for mean and standard deviation is

$$\overline{Q}_m = \sum_{i=1}^{m} \frac{\widehat{Q}}{m}$$

The confidence intervals for the means are

$$\bar{Q} \pm t_{v\ (\alpha/2)}\sqrt{T}$$

where $\sqrt{T}$ is the pooled standard error and $t_{v\ (\alpha/2)}$ is the reference *t* distribution with the degrees of freedom

$$v = (m-1)(1 + r_m^{-1})^2$$

The r_m is the relative increase in variance i.e.

$$r_m = \frac{(\mathrm{B} + \frac{\mathrm{B}}{\mathrm{m}})}{\mathrm{U}}$$

which SPSS reports automatically.

Supporting Methods

References

Eekhout I, De Vet H, Twisk J, Brand J, De Boer M, Heymans M (2014) Missing data in a multi-item instrument were best handled by multiple imputation at the item score level. Journal of Clinical Epidemiology 67:335-342.

Van Ginkel J, Linting M, Rippe R, Van Der Voort A (2019) Rebutting Existing Misconceptions About Multiple Imputation as a Method for Handling Missing Data. *Journal of Personality Assessment* 102:297-308.

Graham J (2009) Missing Data Analysis: Making It Work in the Real World. *Annual Review of Psychology* 60:549-576.

Graham J, Olchowski A, Gilreath T (2007) How many imputations are really needed? Some practical clarifications of multiple imputation theory. *Prevention Science* 8:206-213.

Grigsby T and Mclawhorn J (2019) Missing Data Techniques and the Statistical Conclusion Validity of Survey-Based Alcohol and Drug Use Research Studies: A Review and Comment on Reproducibility. *Journal of Drug Issues* 49:44-56.

Harel O, Mitchell E, Perkins N, Cole S, Tchetgen Tchetgen E, Sun B, Schisterman E (2018) Multiple Imputation for Incomplete Data in Epidemiologic Studies. *American Journal of Epidemiology* 187:576-584.

Karangwa I, Kotze D, Blignaut R (2016) Multiple imputation of unordered categorical missing data: A comparison of the multivariate normal imputation and multiple imputation by chained equations. *Brazilian Journal of Probability and Statistics* 30:521-539.

Kleinke K (2017) Multiple Imputation Under Violated Distributional Assumptions: A Systematic Evaluation of the Assumed Robustness of Predictive Mean Matching. *Journal of Educational and Behavioral Statistics* 42:371-404.

De Silva A, Moreno-Betancur M, De Livera A, Lee K, Simpson J (2019) Multiple imputation methods for handling missing values in a longitudinal categorical variable with restrictions on transitions over time: A simulation study. *BMC Medical Research Methodology*. 19:14.

Supporting Methods

Table SM1. Details of the imputation models. Variables, their level of missingness, and role in imputation models are listed in the order in which they were included in these models. In the main analysis, multiple imputation in item score level was done, but for the sensitivity analysis, multiple imputation in the total score level was used. Differences between these two models are indicated in bold. The variables that were calculated after multiple imputation are listed at the end of the table.

	**Multiple Imputation models**			**Item score level**		**Total score level**	
**N:o**	**Variable**	**Measurement level**	**% missing**	**Role in the model**	**Note**	**Role in the model**	**Note**
1	Pregnancy_duration	Scale	0.00	PRED ONLY		PRED ONLY	
2	Year_of_birth_**Baby**	Scale	0.00	PRED ONLY		PRED ONLY	
3	Sequential_pregnancy_count_Woman	Scale	0.00	PRED ONLY		PRED ONLY	
4	Gravidity_Woman	Scale	0.00	PRED ONLY		PRED ONLY	
5	Parity_Woman	Scale	0.00	PRED ONLY		PRED ONLY	
6	Age_Woman	Scale	0.00	PRED ONLY		PRED ONLY	
7	ART_used_Woman	Nominal	0.00	PRED ONLY		PRED ONLY	
8	Born_alive_**Baby**	Nominal	0.00	PRED ONLY		PRED ONLY	
9	Malformation_**Baby**	Nominal	0.00	PRED ONLY		PRED ONLY	
10	Sex_**Baby**	Nominal	0.00	PRED ONLY		PRED ONLY	
11	Born_in_hospital_**Baby**	Nominal	0.02	IMPUTED		IMPUTED	
12	Postnatal_special_treatment_**Baby**	Nominal	0.00	PRED ONLY		PRED ONLY	
13	Postnatal_days_in_ward_**Baby**	Scale	0.00	PRED ONLY		PRED ONLY	
14	Smoking_status_Woman	Nominal	0.00	PRED ONLY		PRED ONLY	
15	Smoking_status_Partner	Nominal	0.00	PRED ONLY		PRED ONLY	
16	Quit_smoking_DP_Woman	Nominal	0.00	PRED ONLY		PRED ONLY	
17	BP_nicotine_dependency_Woman	Scale	19.61	**PRED ONLY**		**IMPUTED** [a]	

Supporting Methods

	Multiple Imputation models			Item score level		Total score level	
		Measurement	%	Role in the		Role in the	
N:o	Variable	level	missing	model	Note	model	Note
18	DP_nicotine_dependency_Woman	Scale	15.21	**PRED ONLY**		**IMPUTED** [a]	
19	BP_nicotine_dependency_Partner	Scale	14.37	**PRED ONLY**		**IMPUTED** [a]	
20	DP_nicotine_dependency_Partner	Scale	7.91	**PRED ONLY**		**IMPUTED** [a]	
21	Sigma_AUDIT_score_Woman	Scale	17.81	PRED ONLY		PRED ONLY	
22	AUDIT_total_Woman	Scale	27.99	**PRED ONLY**		**IMPUTED** [a]	
23	Sigma_AUDIT_score_Partner	Scale	25.35	PRED ONLY		PRED ONLY	
24	AUDIT_total_Partner	Scale	34.75	**PRED ONLY**		**IMPUTED** [a]	
25	AUDIT_total_missing_Woman	Scale	0.00	PRED ONLY		PRED ONLY	
26	AUDIT_total_missing_Partner	Scale	0.00	PRED ONLY		PRED ONLY	
27	Apgar_mean_**Baby**	Scale	0.01	IMPUTED		IMPUTED	
28	Birthweight_ **Baby**	Scale	0.04	IMPUTED		IMPUTED	
29	Marital_status_Woman	Nominal	0.62	IMPUTED		IMPUTED	
30	Umbilical_cord_length_**Baby**	Scale	1.09	IMPUTED		IMPUTED	
31	Post_membrane_weight_**Baby**	Scale	1.36	IMPUTED		IMPUTED	
32	Number_of_maternity_care_visits_DP_Woman	Scale	3.45	IMPUTED		IMPUTED	
33	Head_circumference_**Baby**	Scale	4.21	IMPUTED		IMPUTED	
34	Head_circumference_Birthweight_ratio_**Baby**	Scale	4.21	PRED ONLY	CALC	PRED ONLY	CALC
35	Height_Woman	Scale	1.41	IMPUTED		IMPUTED	
36	Pregnancy_weight_Woman	Scale	4.51	IMPUTED		IMPUTED	
37	BMI_Woman	Scale	4.56	**PRED ONLY**		**IMPUTED** [a]	
38	Number_of_daily_cigarettes_DP_Partner	Scale	5.79	IMPUTED		IMPUTED	

Supporting Methods

N:o	Multiple Imputation models<br>Variable	Measurement level	% missing	Item score level<br>Role in the model	Note	Total score level<br>Role in the model	Note
39	Number_of_daily_cigarettes_BP_Woman	Scale	6.01	IMPUTED		IMPUTED	
40	First_cigarette_for_HSI_score_DP_Partner	Ordinal	6.96	**IMPUTED**		**PRED ONLY**	
41	Number_of_daily_cigarettes_BP_Partner	Scale	8.47	IMPUTED		IMPUTED	
42	First_cigarette_for_HSI_score_BP_Partner	Ordinal	10.02	**IMPUTED**		**PRED ONLY**	
43	Number_of_daily_cigarettes_DP_Woman	Scale	13.79	IMPUTED		IMPUTED	
44	First_cigarette_for_HSI_score_DP_Woman	Ordinal	14.42	**IMPUTED**		**PRED ONLY**	
45	Age_Partner	Scale	18.34	IMPUTED		IMPUTED	
46	First_cigarette_for_HSI_score_BP_Woman	Ordinal	18.88	**IMPUTED**		**PRED ONLY**	
47	Frequency_of_drinking_DP_Partner	Ordinal	19.10	IMPUTED		IMPUTED	
48	Number_of_maternity_polyclinic_visits_DP_Woman	Scale	18.47	IMPUTED		IMPUTED	
49	AUDITq6_needs_drink_first in the_morning_Woman	Ordinal	19.32	**IMPUTED**		**PRED ONLY**	
50	AUDITq10_someone_concerned_Woman	Ordinal	19.39	**IMPUTED**		**PRED ONLY**	
51	AUDITq4_unable_to_stop_Woman	Ordinal	19.41	**IMPUTED**		**PRED ONLY**	
52	AUDITq5_failed_duties_Woman	Ordinal	19.46	**IMPUTED**		**PRED ONLY**	
53	AUDITq9_alc_caused_injuries_Woman	Ordinal	19.60	**IMPUTED**		**PRED ONLY**	
54	AUDITq8_unable_to_remember_Woman	Ordinal	19.59	**IMPUTED**		**PRED ONLY**	
55	AUDITq7_feeling_guilt_Woman	Ordinal	19.71	**IMPUTED**		**PRED ONLY**	
56	AUDITq3_freq_of_binge_drinking_Woman	Ordinal	19.74	IMPUTED		IMPUTED	
57	AUDITq2_typical_number_of_drinks_Woman	Ordinal	19.96	**IMPUTED**		**PRED ONLY**	
58	AUDITq1_freq_of_drinking_Woman	Ordinal	23.90	IMPUTED		IMPUTED	
59	Number_of_weekly_drinks_DP_Partner	Scale	21.53	IMPUTED		IMPUTED	

Supporting Methods

	Multiple Imputation models			Item score level		Total score level	
N:o	Variable	Measurement level	% missing	Role in the model	Note	Role in the model	Note
60	Frequency_of_drinking_DP_Woman	Ordinal	9.94	IMPUTED		IMPUTED	
61	Number_of_weekly_drinks_DP_Woman	Scale	9.39	IMPUTED		IMPUTED	
62	AUDITq2_typical_number_of_drinks_Woman	Ordinal	27.56	**IMPUTED**		**PRED ONLY**	
63	AUDITq6_needs_drink_firstinthemorning_Woman	Ordinal	27.80	**IMPUTED**		**PRED ONLY**	
64	AUDITq3_freq_of_binge_drinking_Woman	Ordinal	27.74	IMPUTED		IMPUTED	
65	AUDITq4_unable_to_stop_Woman	Ordinal	27.80	**IMPUTED**		**PRED ONLY**	
66	AUDITq10_someone_concerned_Woman	Ordinal	27.83	**IMPUTED**		**PRED ONLY**	
67	AUDITq5_failed_duties_Woman	Ordinal	27.90	**IMPUTED**		**PRED ONLY**	
68	AUDITq9_alc_caused_injuries_Woman	Ordinal	28.13	**IMPUTED**		**PRED ONLY**	
69	AUDITq8_unable_to_remember_Woman	Ordinal	28.16	**IMPUTED**		**PRED ONLY**	
70	AUDITq7_feeling_guilt_Woman	Ordinal	28.28	**IMPUTED**		**PRED ONLY**	
71	AUDITq1_freq_of_drinking_Woman	Ordinal	29.39	IMPUTED		IMPUTED	
72	Self_reported_drinking_category_DP_Partner	Ordinal	0.00	IMPUTED		IMPUTED	
73	Self_reported_drinking_category_DP_Woman	Ordinal	0.00	IMPUTED		IMPUTED	
74	Number_of_weekly_drinks_BP_Woman	Scale	42.52	IMPUTED		IMPUTED	
75	Number_of_weekly_drinks_BP_Partner	Scale	48.36	IMPUTED		IMPUTED	
#1	AUDIT_score_total_afterMI_Woman	Scale	-	-	**CALC**	-	a
#2	AUDIT_score_total_afterMI_Partner	Scale	-	-	**CALC**	-	a
#3	BMI_afterMI_Woman	Scale	-	-	**CALC**	-	a
#4	BP_daily_cigarette_score_Partner	Ordinal	-	-	**CALC**	-	b
#5	DP_daily_cigarette_score_Partner	Ordinal	-	-	**CALC**	-	b

Supporting Methods

	Multiple Imputation models			Item score level		Total score level	
		Measurement	%	Role in the		Role in the	
N:o	Variable	level	missing	model	Note	model	Note
#6	BP_daily_cigarette_score_Woman	Ordinal	-	-	CALC	-	b
#7	DP_daily_cigarette_score_Woman	Ordinal	-	-	CALC	-	b
#8	BP_nicotine_dependency_score_Partner	Scale	-	-	CALC	-	a
#9	DP_nicotine_dependency_score_Partner	Scale	-	-	CALC	-	a
#10	BP_nicotine_dependency_score_Woman	Scale	-	-	CALC	-	a
#11	DP_nicotine_dependency_score_Woman	Scale	-	-	CALC	-	a

Abbreviations; AUDITq1: Alcohol Use Disorder Identification Test Question number 1; BMI: Body Mass Index; BP: Before Pregnancy; CALC: Variable calculated after multiple imputation; DP: During Pregnancy; IMPUTED: Variable imputed multiple times; MI: Multiple Imputation; PRED ONLY: Variable not imputed, but used as a predictor only. These #1–#11 score variables not calculated because [a] the total score variables of the model multiple imputed and thus [b] these scores are not needed.
