# Supplementary material for: Self‐reported alcohol consumption of pregnant women and their partners correlates both before and during pregnancy: A cohort study with 21,472 singleton pregnancies
Source: Alcohol Clin Exp Res. 2022 May 15;46(5):797–808. doi: 10.1111/acer.14806 (PMC9321706; doi:10.1111/acer.14806)
Supplement: Supplementary file 9 — Supplementary Material [file ACER-46-797-s009.docx]

**Supporting Information Legends**

**Supporting Methods. Multiple Imputation (MI).** Includes the justification for the use of MI, an example of the SPSS Syntax code, used formulas for calculating the multiple imputed pooled results, and a table specifying the two imputation models. In the main analysis, multiple imputation in the item score level was done, but in the sensitivity analysis, multiple imputation in the total score level was used. The table shows the variables, their level of missingness, and role in imputation models. The variables are listed in the order where they were in these models and differences between the two imputation models are indicated in bold. Variables that were calculated after multiple imputation are listed at the end of the table.

**Table S1. The mean and standard deviations of the variables in the original data.** A red bold font indicates where the estimates of the original data differ from those obtained from item score level multiple imputed estimates.

**Table S2. Sensitivity analysis of the multiple imputed estimates.** Differences between the mean and standard deviations of the item and total score level multiple imputed variables are highlighted in red. Due to the great similarity of the estimates, no age division was made for women and their partners.

**Table S3. Sensitivity analysis for independent cases.** N = 14 822 women. For independent case analysis one pregnancy was randomly selected for each woman in the cohort. Red bold font indicates the level where the estimates of the randomly selected one pregnancy per women sample (n = 14 822) differed from results obtained from data including all 21 472 pregnancies.

**Figure S1. Correlation heatmap of the self-reported alcohol use and the age variables of the women and their partners in the original data.** Statistically significant correlations (p < 0.0005) are indicated in bold. The valid number of cases (**n**) in each correlation is indicated below the Spearman rho’s correlation coefficient (**r_s_**). N = 21 472. The grey rectangles indicate correlations where the correlation was statistically significant in the original data but not in the multiple imputed data, and the green rectangles indicate correlations where the correlation was statistically significant in the multiple imputed data but not in the original data. A black font in the correlation coefficients indicates that the difference between the original and the multiple imputed correlation coefficients differed more or equal to 0.15, while the blue points to a difference smaller than that value. **^a^** The before-pregnancy frequency of drinking and the frequency of binge drinking are questions in the AUDIT questionnaire.

**Figure S2. Sensitivity analysis of the correlation coefficients**. The Spearman rho’s correlation coefficients of alcohol use and age variables of the women and their partners are those of the total score level multiple imputed data (MI = 40). Thus, the N in each cell is 21 472. Statistically significant correlations (p < 0.0005) are indicated in bold text. There were no differences in the statistical significance of the correlation coefficients between the two multiple imputations. The differences in the coefficients are indicated with blue font. **^a^** The before-pregnancy frequency of drinking and frequency of binge drinking are both questions in the AUDIT questionnaire, which explains the stronger correlation between them and the total AUDIT score than the rest of the variables.

**Figure S3. Sensitivity analysis for the independent cases frequency data.** N = 14 822 women. For independent case analysis one pregnancy was randomly selected for each woman in the cohort. The bar graphs represent the women’s **A** frequency of drinking before pregnancy (n of valid cases i.e., the number of women having answered to the question was 11 292); **B** frequency of binge drinking before pregnancy (n of valid cases 11 906); **C** frequency of drinking during pregnancy (n of valid cases 9729); and **D** alcohol use status during pregnancy (n of valid cases 13 472). Note that multiple imputation (MI) cannot be used to report frequencies. Thus, the frequency of all cases bar graphs show the proportion of women in each age group having a missing answer in the original data and the frequency of valid cases bar graphs illustrates the distribution of the valid answers in each age group (each age group gives a total of 100%) in the original data. Note, that the most important general comparisons stated under these graphs were similar in both the independent sample and nonindependent cohort (n = 21 472 pregnancies). This indicates that the results of our nonindependent pregnancies (i.e. proportion of pregnancies) can be generalized to proportion of women in our cohort. For example, in A in the independent sample in total 87.4% of women reported using alcohol before pregnancy whereas in the whole cohort women did so in 85.7% of the pregnancies. Compare panels **A** & **B** to **Fig. 3 A & C**, respectively, and panels **C** & **D** to **Fig. 4 A & C**, respectively.

**Figure S4. Sensitivity analysis for the independent cases correlation coefficient.** N = 14 822 women. For independent case analysis one pregnancy for each woman in the cohort was randomly selected. These coefficients were compared to those obtained from analyzing all the pregnancies in the cohort (n = 21 472 pregnancies) (see **Figure S1.**). Coefficients in these two analyses were similar (i.e., the differences were less than 0.05), there were no differences in their direction, and no major differences in their statistical significances. Thus, although our cohort deviated from the assumption of independence, this has very little effect on results and did not affect the interpretation of the results. Statistically significant correlations (p < 0.0005) are indicated in bold. The valid number of cases (n) in each correlation is indicated below the Spearman rho’s correlation coefficient (r_s_). **^a^** The before-pregnancy frequency of drinking and the frequency of binge drinking are questions in the AUDIT questionnaire.
